# Structure Sensitivity and Catalyst Restructuring for CO_2_ Electro-reduction on Copper

**DOI:** 10.1038/s41467-025-59267-3

**Published:** 2025-04-30

**Authors:** Dongfang Cheng, Khanh-Ly C. Nguyen, Vaidish Sumaria, Ziyang Wei, Zisheng Zhang, Winston Gee, Yichen Li, Carlos G. Morales-Guio, Markus Heyde, Beatriz Roldan Cuenya, Anastassia N. Alexandrova, Philippe Sautet

**Affiliations:** 1 Department of Chemical and Biomolecular Engineering, University of California, Los Angeles, CA USA; 2 Department of Interface Science, Fritz Haber Institute of the Max Planck Society, Faradayweg 4-6, Berlin, Germany; 3 Department of Chemistry and Biochemistry, University of California, Los Angeles, CA USA; 4 Department of Mechanical and Aerospace Engineering, Princeton University, Princeton, NJ USA; 5 Department of Materials Science and Engineering, University of California, Los Angeles, CA USA; 6 California NanoSystems Institute, Los Angeles, CA USA

**Keywords:** Electrocatalysis, Density functional theory

## Abstract

Cu is the most promising metal catalyst for CO_2_ electroreduction (CO_2_RR) to multi-carbon products, yet the structure sensitivity of the reaction and the stability versus restructuring of the catalyst surface under reaction conditions remain controversial. Here, atomic scale simulations of surface energies and reaction pathway kinetics supported by experimental evidence unveil that CO_2_RR does not take place on perfect planar Cu(111) and Cu(100) surfaces but rather on steps or kinks. These planar surfaces tend to restructure in reaction conditions to the active stepped surfaces, with the strong binding of CO on defective sites acting as a thermodynamic driving force. Notably, we identify that the square motifs adjacent to defects, not the defects themselves, as the active sites for CO_2_RR via synergistic effect. We evaluate these mechanisms against experiments of CO_2_RR on ultra-high vacuum-prepared ultraclean Cu surfaces, uncovering the crucial role of step-edge orientation in steering selectivity. Overall, our study refines the structural sensitivity of CO_2_RR on Cu at the atomic level, highlights the self-activation mechanism and elucidates the origin of in situ restructuring of Cu surfaces during the reaction.

## Abstract (web summary)

Atomic-scale simulations and microscopy experiments reveal copper surfaces restructure during CO_2_ electroreduction, driven by CO adsorption, forming active sites adjacent to defects that are crucial for catalyst activity and product selectivity.

## Introduction

Numerous studies, both experimental and theoretical, have reported that the product distribution in electrochemical CO_2_ reduction (CO_2_RR) is modified when changing the surface termination of Cu^1–8^. Hori et al. observed that CH_4_ production is dominant among the hydrocarbon products on Cu(111) and Cu(110) surfaces, whereas Cu(100) yields a higher amount of C_2_H_4_ products compared to C1 products^1^. Interestingly, the inclusion of (100) steps on (111) terrace sites did not significantly increase the selectivity of C_2_ products generation; in contrast, the incorporation of (111) or (110) steps on these (111) terrace surfaces improved the faradaic efficiency for C_2_H_4_ over CH_4_, indicating that specific step sites can have a positive effect on the generation of C-C coupling products. Notably, the introduction of optimal step sites to Cu(100) resulted in an increment in C_2_ products production. Hahn et al. synthesized Cu(751) films with abundant kinked sites and found that the selectivity for C2 products, particularly alcohols, can be enhanced compared to those on the planar Cu(111) and Cu(100) surfaces^3^. The collected information suggests a correlation between the density of undercoordinated sites and the selectivity for C_2_ products. Nonetheless, it should be considered that the majority of the prior work reporting a facet-sensitivity for CO_2_RR was conducted on Cu single crystals previously exposed to an electropolishing treatment that drastically affected the surface structure and led to an increased defect density, rendering experimental surfaces very different from the ones theoretically modeled. So far, only two studies have been carried out on Cu(100) and Cu(111) single crystal surfaces prepared in ultra-high vacuum (UHV)^9,10^, and such work revealed that the flat and clean (non-electropolished) surfaces were characterized by a facet-independent selectivity towards hydrogen evolution. In fact, only when defects were introduced by different external treatments or the reaction environment itself could hydrocarbons be generated.

Thus, the current portrayal of facet-dependent activity in Cu-based catalysts during CO_2_RR needs to be revisited in order to gain an in-depth understanding of the structure-performance relationship of these catalysts. Particularly, two significant issues must be emphasized and addressed. Firstly, mechanically and electrochemically polished electrodes with varying surface orientations cannot be classified as true single-crystal surfaces, as they possess multiple steps and defects. Scholten et al. prepared well-ordered atomically clean Cu single crystals (111) and (100) surfaces under UHV conditions and observed a strong preference for the hydrogen evolution reaction versus CO_2_RR, which yielded a very small fraction of hydrocarbon products. It was only on the surfaces prepared with steps and other defects that C_2_ products were generated^9^. Nguyen et al.’s work further emphasizes the importance of step edges and kink sites, showing on UHV-prepared Cu(111) single crystals that the step density increased as a function of the number of CO_2_RR and subsequent UHV treatments, which overall gives rise to an increase in hydrocarbon production^10^. Another example is that Kim et al. did not detect any ethanol on the atomically ordered Cu(100) surface during COR^11^. The fact that the Cu(100) film synthesized by Hahn et al. show just one order of magnitude higher activity for C_2_ products compared to their Cu(111) surface, contradicts computational predictions that Cu(100) is markedly superior to Cu(111) in terms of C_2_ product formation, since C-C coupling barriers are 0.3-0.5 eV higher on Cu(111) than on Cu(100)^4,8,12^, which should provide a five orders of magnitude lower rate on Cu(111). This indicates again that the measured reactivity on Cu(111) is not intrinsic but likely governed by defects and that the type and density of those defects might also strongly depend on the specific reaction conditions, and sample preparation history, and prior use.

In addition, a number of cutting-edge techniques have demonstrated that the Cu surface undergoes significant restructuring when subjected to CO_2_RR conditions, even when the surface is initially well-defined, with a low density of defects. Soriaga et al. employed quasi-*operando* electrochemical scanning tunneling microscopy (ECSTM) and differential electrochemical mass spectrometry to illustrate that Cu(100) undergoes a transformation to Cu(S)-[3(100)×(111)] and that such restructuring will lead to the ethanol production^11^. Grosse et al. tracked the dynamic evolution of Cu nanocube catalyst through real-time electrochemical liquid cell transmission electron microscopy (ECTEM) and showed that cubic-shape Cu_2_O catalysts restructure from a solid single crystalline form to a fragmented nanoporous structure under CO_2_RR conditions, with some small randomly shaped NPs being leached out from cubes^13^. Moreover, the former authors were able to correlate the evolving restructuring of their Cu catalysts with drastic modification of their product selectivity, in particular, C_2_H_4_ formation. Buonsanti and Marzari et al. proposed a potential-driven nanoclustering degradation mechanism in Cu nanocubes (CuNCs) during CO_2_RR, where the electrode potential is the driving force to degrade the CuNCs nanocubes^14^. Unwin et al. employed scanning electrochemical cell microscopy (SECCM) together with co-located electron backscatter diffraction (EBSD) as a screening technique to demonstrate that electroreduction activity scales with the step and kink density of the surfaces^15^. Goddard, Huang, and colleagues presented findings on the self-activation of Cu nanowires, transitioning from (100) surfaces to stepped surfaces, and H adsorption was identified as the driving force for the restructuring^16^. In addition, Yang et al. used EC-STEM and 4D-STEM to study the structural dynamics and suggested that Cu nanoparticles will evolve into metallic Cu nanograins, with undercoordinated active sites on the nanograins being responsible for C-C coupling^17^. Jiang and colleagues simulated the plasma treatment and Ar^+^ bombardment roughening of a Cu(111) surface, resulting in the formation of square motifs near under-coordinated sites, which they identified as the active sites for C2 product generation^18^. Very recent work from Magnussen et al. using electrochemical scanning tunneling microscopy (EC-STM) also unveiled the formation of small Cu clusters on Cu(100) during CO_2_RR and discussed their role in the product selectivity^19^.

The experimental results indicate that Cu surfaces undergo restructuring under CO_2_RR conditions^19^, although obtaining detailed atomic structures from experimental methods proves to be challenging. These findings raise several pertinent questions: (1) What is the driving force for surface restructuring during reduction conditions? (2) Why is the atomically ordered clean Cu(100) surface not active for C_2_ products, despite extensive computational evidence supporting its role as a good active site for C-C coupling? (3) Is the C-C coupling reaction barrier calculated from DFT the sole determinant of C_2_ activity in CO_2_RR, or are other factors necessary to fully characterize this behavior?

Herein, the theoretical approach employed grand canonical density functional theory (GCDFT) calculations and kinetic analysis based on the ensembles obtained from global optimization to investigate the role of defected sites, and to demonstrate the lack of CO_2_RR on planar Cu surfaces. We developed a random phase approximation (RPA) site-preference corrected grand canonical basin hopping (GCBH) method to sample the chemical space of CO on various Cu surfaces, including planar surfaces and different types of steps and kinks. Our results emphasized that planar Cu(111) and Cu(100) surfaces exhibit extremely low CO coverage due to both sluggish CO_2_ conversion and unfavorable CO binding, leading to almost no CO_2_RR activity to multi-carbon products on these surfaces, as confirmed by a kinetic analysis. Conversely, steps and kinks contribute the most to CO_2_RR activity, despite not exhibiting decreased C-C coupling barriers, suggesting that commonly calculated C-C coupling barriers are not the sole factor for determining the CO_2_RR activity. Our findings suggest that commonly used Cu(111) and Cu(100) surfaces are not appropriate models for CO_2_RR studies in theory, as they are inactive for CO_2_RR. We demonstrate that specific motifs, formed by a square arrangement of Cu atoms and associated with steps, kinks, or other defective sites, are the key to CO_2_RR towards C_2_ products. Furthermore, the strong CO binding on steps and kinks drives the in situ formation of step and kink sites from clean planar surfaces, resulting in restructuring under CO_2_RR reactive conditions. Such restructured stepped surfaces, created during the reaction, play a crucial role in generating the active sites for CO_2_RR. Our modeling data is validated against microcopy images of well-defined planar and stepped single crystal Cu surfaces and corresponding CO_2_RR selectivity data.

## Results

### Structure sensitivity of CO adsorption on Cu surfaces

CO is a central intermediate in CO_2_RR on Cu, Au, and Ag surfaces. Therefore, we first focus on the structure sensitivity for CO adsorption on various Cu surface terminations under realistic potentials. We specifically selected planar surfaces, namely Cu(111) and Cu(100), as well as surfaces with different terminations and steps. These include Cu(711), which consists of (100) terrace with (111) steps, Cu(533), comprising (111) terrace with (100) steps, Cu(430) with (110) terrace and (111) steps, and Cu(410) featuring (100) terrace with (110) steps. Additionally, we considered surfaces with kinked sites, such as Cu(843), comprising (111) terrace with (100) steps and kinks, and Cu(1021), consisting of (100) terrace with (111) steps and kinks (Supplementary Fig. 1, 2). By incorporating these varieties of surfaces, we aim to encompass a broad spectrum of local sites that could potentially influence CO adsorption and further reactivity on Cu surfaces.

Considering the large number of possible CO coverages and for each coverage the extensive number of configurations, we employ the grand canonical basin hopping (GCBH) method to globally optimize the density and structure of the CO adlayer on the eight distinct Cu surfaces. In the GCBH approach^20–22^, the system is allowed to exchange CO with a reservoir with a fixed CO chemical potential, *μ*_CO_(*P*, *T*). To mimic the experimental conditions for CO_2_RR, GCBH samplings are performed at room temperature and at a CO pressure of 0.05 atm, which corresponds to the CO partial pressure observed during CO_2_RR on the polycrystalline Cu surface^23^. It should be noted that in this step, we assume that these surfaces do not restructure but remain static upon CO adsorption. A substantial number of structures, approximately ~ 1000, were sampled for each surface, spanning a range of CO coverages and binding configurations. The purpose of this extensive sampling was to capture the diversity of possible adsorption states and their associated energies. Subsequently, grand canonical density functional theory (GCDFT)^24–27^ is employed to polarize the surface and to determine the influence of the electrode potential on the global minima (GM) and low energy metastable ensembles (LEME) from the GCBH dataset, resulting in a grand canonical ensemble representation of both the geometric structure and the energy at the given electrode potential.

It is well known that semi-local density functionals tend to underestimate the HOMO-LUMO gap for CO and place the unfilled CO 2π* orbital too low in energy^28,29^. Consequently, this results in an artificial strengthening of the 2π*-*d* back-bonding interactions, which favors multiply bonded sites in an unphysical manner. As a result, semi-local density functionals fall short in describing CO adsorption energies and, in particular, the binding site preference on several metal surfaces, which is commonly known as the ‘CO adsorption puzzle’.

**Fig. 1:**
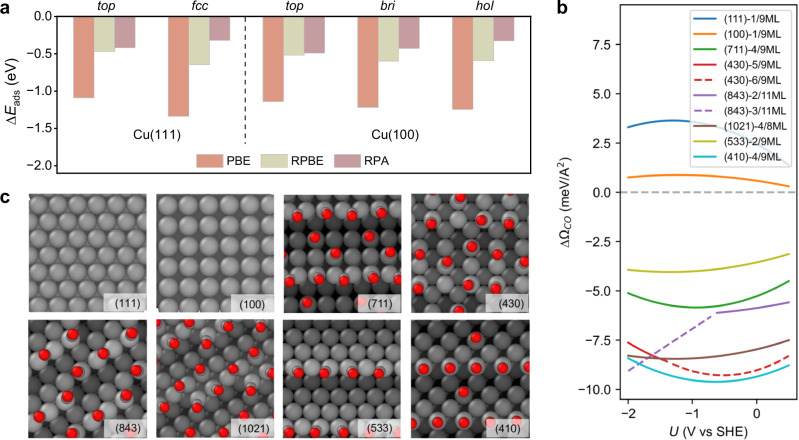
Structure sensitivity of CO adsorption. **a** Comparison of adsorption energy of CO on different sites at Cu(111) and Cu(100) using RPA and GGA level PBE/RPBE functionals. RPA results for Cu(111) are from Kresse et al.^30^. **b** The surface area normalized adsorption free energy as a function of the electrode potential. The GM structures for each coverage are obtained with the combined RPA-corrected site preference global optimization and grand canonical DFT. The optimal coverages for 8 different Cu surfaces under the CO pressure of 0.05 atm are shown. For Cu(430) and Cu(843), the consecutive solid and dotted lines indicate a change of CO coverage as a function of potential. **c** GM structures of CO adsorption arrangements for 8 different Cu surfaces under the same condition and a potential of − 1.5 V vs SHE.

To address these limitations, we utilized the random phase approximation (RPA), a post-Hartree-Fock method based on many-body perturbation theory, to explore the CO adsorption on Cu(111) and Cu(100) surfaces^30–33^. Our finding revealed that on both Cu(111) and Cu(100) surfaces, the PBE functional significantly overestimates the binding strength of CO, which will lead to unrealistically high coverage on the planar Cu(111) or Cu(100) surface. Furthermore, PBE and RPBE functionals exhibit a preference for CO adsorption on the *hollow* site or *bridge/hollow* site, on Cu(111) and Cu(100), respectively, whereas RPA favors the *top* site for both surfaces (Fig. 1a). These CO binding preferences align with experimental results and other advanced computational methods, such as embedded correlated wavefunction theory^34^ and doubly hybrid XYG3 functional^35^. Notably, despite the limitation of the RPBE functional in accurately describing CO binding on *hollow* sites due to its tendency to excessively bind CO on such sites, it exhibits a similar CO binding energy value as RPA for *top* site adsorption.

Taking this into consideration, we developed a global optimization approach called the RPA site-preference corrected grand canonical basin hopping (GCBH) method. This method allows us to explore the chemical space, encompassing different binding configurations and coverages of CO on Cu surfaces using the RPBE functional, where CO is constrained to the *top* site on the terrace, thus incorporating considerations of both energy accuracy and site preference. At a pressure of 0.05 atm, our results show that the Cu(111) and Cu(100) surfaces do not exhibit CO adsorption (Fig. 1b). The calculated adsorption free energies for CO on these surfaces are positive over a wide range of potentials. In contrast, it is observed that steps and kinks on Cu surfaces have strong binding affinity toward CO, resulting in nearly full coverage of CO. This was also featured in the work by Scholten et al. that displayed a stronger CO desorption temperature in temperature-programmed experiments for Cu(111) and Cu(100) surfaces with a higher density of step steps as compared to pristine (UHV-prepared) analogous surfaces^9^. In most cases, CO exhibits *top*-*bridge*-*top* adsorption arrangement on these defective sites (Fig. 1c). Among the various terrace and step/kink configurations investigated, the (110) termination, either in the form of a terrace or containing steps, such as Cu(410) and Cu(430), exhibits stronger CO binding compared to the other terrace or step sites. This finding is in agreement with the work by Nguyen et al., where cyclic voltammetry measurements display peaks assigned to Cu(110) surface features on the hydrocarbon producing UHV-prepared Cu(111) surface, hinting that these (110) sites are probably linked to the CO_2_RR active sites^10^. Furthermore, in the potential range of interest, specifically at potentials more negative than − 1 V vs SHE, surfaces with additional kinked sites, such as Cu(843) and Cu(1021), show significantly stronger CO binding compared to Cu(533) and Cu(711), which have similar termination but lack such kink sites. It is worth noting that these steps and kink sites will not only display enhanced CO binding themselves, but also influence the coordination environment in their vicinity, namely, providing lower generalized coordination numbers^36,37^. Consequently, some CO molecules may also adsorb onto adjacent sites near the steps and kinks, even in the presence of adjacent planar (111) and (100) terminations where CO adsorption is not expected, which leads to notably higher CO coverages in the step and kink region. Moreover, an absence of CO adsorption is observed at the bottom of steps and kinks, attributable to both the presence of highly coordinated Cu atoms and steric hindrance for CO adsorption. In short, step and kink sites, along with their neighboring regions, exhibit significantly stronger CO binding and substantially higher CO coverage compared to planar Cu surfaces. For the cases under different CO pressures, see Supplementary Fig. 3–5.

In the framework of GCDFT, the potential-dependent adsorption energy of CO (∆Ω_*i,CO*_(*U*)) is a quadratic function of *U*, whose characteristic is determined by the change of Helmholtz capacitance (∆*C*_H_) and the change of potential of zero charge (∆*pzc*) upon CO adsorption. Notably, distinct surfaces demonstrate diverse dependencies, characterized by different curvatures and slopes, with respect to CO adsorption. The curvature of this quadratic relationship is solely determined by ∆*C*_H_, while the slope of the tangent line at a given potential$\,\left(\frac{\partial \Delta {\Omega }_{i,{CO}}(U)}{\partial U}\right)$ on the curve is determined by ∆*C*_H_, ∆*pzc*, and specific *U* at this point. Therefore, the distinct characteristics observed for different surfaces in Fig. 1b can be attributed to the varying degrees of change in the *pzc* and *C*_H_ upon CO adsorption. For details, see Supplementary Note 1.

### Stability of Cu surfaces under optimum CO coverage: thermodynamic restructuring trends

**Fig. 2:**
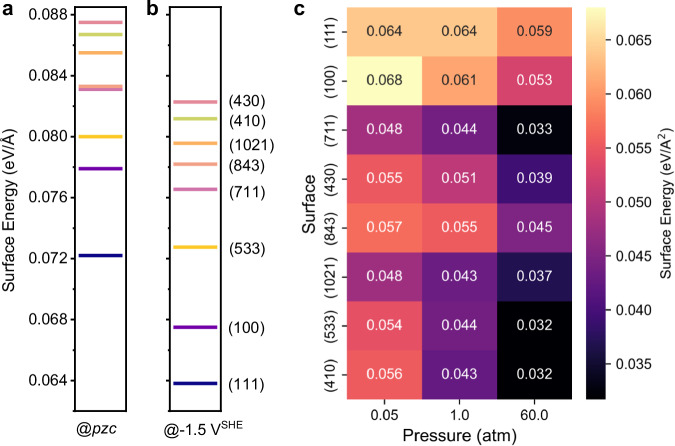
Stability of Cu surfaces under potential and CO pressure. Surface energy of various Cu terminations at (**a**) the potential of zero charge (*pzc*) and (**b**) − 1.5 V vs SHE without CO adsorption. **c** Surface energy as a function of CO pressure for various surfaces at − 1.5 V vs SHE.

The various considered Cu surfaces present different stabilities, as shown by their surface energies. It is well known that in the absence of potential and CO adsorbates, the dense planar surfaces Cu(111) and Cu(100) are the most stable ones (Fig. 2a) and would dominate on a Cu nanoparticle. Stepped and kinked surfaces, with lower coordination surface atoms, are intrinsically less stable. Upon application of a reducing potential (− 1.5 V vs SHE selected for Fig. 2c) to the bare surfaces, in the absence of CO adsorbates, the stability order does not change (Fig. 2b). As the potential becomes more negative than the *pzc*, the surface energies slightly decrease, indicating that the negatively charged surfaces become more stable compared to the uncharged ones (Supplementary Fig. 6).

In the presence of CO adsorption at optimum coverage and under potential, however, this stability trend of Cu surfaces is completely inverted (Fig. 2c). The potential of − 1.5 V vs SHE was chosen as it corresponds to the maximum activity for CO_2_RR. At the CO pressure of 0.05 atm, both Cu(111) and Cu(100) exhibit no CO adsorption, maintaining a clean state and thus resulting in no change in the surface energy. However, a substantial stabilization occurs on the step and kinked surfaces at their optimum CO coverages (Fig. 2c, Supplementary Note 2). All the stepped and kinked surfaces considered in our work demonstrate higher stability compared to Cu(100). Certain surfaces, such as those possessing (110) termination like Cu(410) and Cu(430), as well as kinked surfaces like Cu(843), that in the bare state are the most unstable, exhibit even greater stability than Cu(111) at optimum CO coverage.

Upon increasing the CO pressure to 1 atm, the Cu(111) surface still shows no CO adsorption at equilibrium, while Cu(100) exhibits 1/3 ML CO coverage, although the adsorption strength is relatively weak compared to the step or kink sites. Moreover, at 1 atm CO pressure, the interfacial energies for all the stepped and kinked surfaces are lower than those of Cu(111) and Cu(100). As the CO pressure is further increased, a stabilization effect can be seen on Cu(111) and Cu(100) surfaces since the CO adsorption strength is enhanced and coverages are increased, however, the stepped and kinked surface remain significantly more stable. Hence, it is clear that the stabilities of stepped and kinked surfaces versus planer (111) and (100) show a large sensitivity to CO pressure, particularly at moderate pressures, because of the strong CO adsorption and much higher CO coverage.

The pronounced stabilization of stepped and kinked surfaces under CO has major consequences on the structure of the catalytic Cu surfaces in CO_2_RR reaction conditions: It indicates a strong thermodynamic tendency for the restructuring of planar surfaces toward the formation of steps, kinks, and other defects, driven by the strong CO binding. The restructuring process is influenced by both the electrode potential and the CO partial pressure. Such findings can explain many experimental observations of the fact that the well-prepared Cu surfaces will undergo dramatic restructuring under conditions of CO_2_RR. Such restructuring involves the formation of new Cu facets and hence requires the important transport of Cu atoms at the surface. Hence, such restructuring is not solely governed by thermodynamics, but also influenced by the kinetics of mass transport, thus requiring some time to take place. Obtaining a detailed pathway for such restructuring is highly challenging, goes beyond the scope of this paper, and will be the topic of future studies.

### CO_2_ conversion to CO: low-coordinated sites account

Until this point, we have assumed that CO is formed on various surfaces from CO_2_ electro-reduction. We will now focus on that initial step. An intriguing phenomenon observed by Roldan and co-workers is the dominance of hydrogen evolution reaction (HER) over CO_2_RR on extremely clean Cu(111) and Cu(100) surfaces^9^. Only a small amount of hydrocarbon products is formed, and the estimated CO pressure observed is low, ~ 0.01 atm. The significantly lower CO pressure observed on clean Cu(111) and Cu(100) compared to polycrystalline Cu surfaces suggests a potential difference in the process of CO_2_ conversion to CO between planar and defective surfaces. Motivated by this, we thus investigate the CO_2_ activation on various surfaces.

**Fig. 3:**
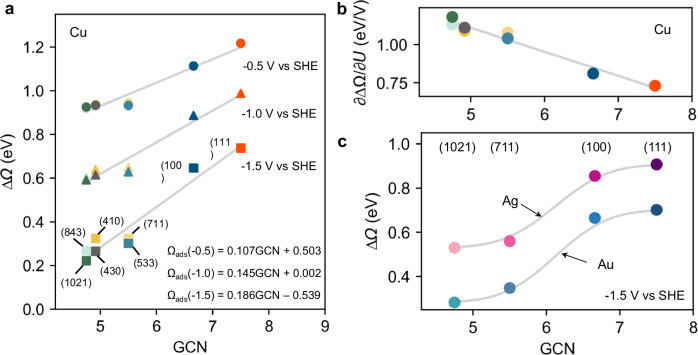
Potential- and facet- dependence of CO_2_ adsorption. **a** The adsorption free energy of CO_2_ (∆Ω) on various surfaces as a function of the general coordination number (GCN) and the electrode potential. **b** The charge transfer coefficients (∂∆Ω/∂*U*) as a function of GCN, here we assume that the relationship between ∆Ω and *U* is linear. **c** The adsorption free energy of CO_2_ (∆Ω) as a function of GCN at − 1.5 V vs SHE on various Au and Ag surfaces.

CO_2_ electroreduction requires its chemisorption and activation as a bent CO_2_ structure on the catalyst. We will then consider the CO_2_ chemisorption free energy barrier as a basic reactivity descriptor. Clearly, if chemisorption is difficult, then the first step of CO_2_RR towards CO will be difficult as well. Since previous studies have shown that the CO_2_ adsorption barrier can be well approximated by the adsorption energy, we here adopt the CO_2_ adsorption free energy as a reactivity descriptor^38^. Our calculations show that the adsorption free energy of CO_2_ exhibits a linear relationship with the general coordination number of the surface Cu atom. Notably, the slopes of this linear relationship vary depending on the electrode potentials, with larger slope values observed at more negative potentials (Fig. 3a and Supplementary Fig. 7). This intriguing trend suggests that the adsorption behavior is influenced by the applied potential, particularly enhancing the differences between planar Cu(111)/(100) surfaces and stepped/kinked surfaces. Specifically, the CO_2_ adsorption energy on Cu(111) and Cu(100) surfaces displays a lower sensitivity to the potential changes compared to that on the stepped and kinked surfaces, which is linked with a lower electron transfer coefficients for the planar surfaces that should high CGN (Fig. 3b). Consequently, a larger structure sensitivity of CO_2_ adsorption can be observed at more negative potentials, which explains the increased slope of the linear relationship with CGN as the potential becomes more negative.

In comparison to step and kink sites, the chemisorption of *CO_2_ is much more endergonic on the Cu(111) and Cu(100) surfaces. Even at − 1.5 V vs SHE, the adsorption free energies for both planar surfaces exceed 0.6 eV, whereas the reaction energies on the step and kink sites are approximately 0.25 eV. The difficult CO_2_ adsorption implies sluggish kinetics for CO_2_ conversion to CO on pure Cu(111) and Cu(100) surfaces, which explains the low CO production observed on planar Cu surfaces in the experiments. The consequent low CO pressures and the moderate CO adsorption energy result in the absence of CO adsorbate on planar Cu surfaces in reaction conditions.

We extend our investigation to the other transition metals, such as Au and Ag, known for their excellent catalytic activity in CO_2_RR to CO. Similarly, we observed that CO_2_ chemisorption is markedly endergonic on planar Au and Ag (111) and (100) surface, even at a highly negative potential (i.e., − 1.5 V vs SHE) typically associated with maximum CO production, with free energy values similar to those on Cu for Au and even more positive for Ag (Fig. 3c and Supplementary Fig. 8). Considering that CO_2_ chemisorption is regarded as the rate-determining step (RDS) on Au and Ag^38^, such endergonic adsorption will result in low activity for CO_2_ to CO, contradicting experimental findings^39^. In contrast, step and kink sites exhibit significantly less endergonic CO_2_ chemisorption, suggesting that on Ag and Au catalysts, these low-coordinated sites, rather than the inert planar (111) and (100) surfaces commonly used in CO_2_RR computational studies, serve as the active site. CO_2_ chemisorption remains endergonic and rate limiting on the step/kink sites, but the cost in free energy to reach the chemisorbed state remains reasonable so that the process can be kinetically accessible at room temperature.

We globally sampled CO adsorption on the various Au and Ag surfaces as well to see if any similarities to the observed restructuring trend on Cu occur. The potential-dependent CO adsorption on Au and Ag reveals that CO adsorption is relatively weak and always exhibits endothermic nature both on planar sites and on step and kink sites in the wide potential range (− 2–0.5 V vs SHE), indicating that the equilibrium coverage of CO on Au and Ag remains near zero, even at defective sites, eliminating the thermodynamic driving force needed for restructuring(Supplementary Figs. 9 and 10). Considering that adsorbed *CO is positioned at the lowest energy in the whole energy span of CO_2_ conversion to CO on Au and Ag, the restructuring cannot be expected to be induced by the other intermediates along the CO_2_RR. This stands in contrast to the behavior observed on Cu, highlighting that planar surfaces of Au and Ag will not demonstrate significant CO_2_RR activity even over prolonged periods of testing, as restructuring is not feasible on these surfaces.

### CO_2_RR to C_2_ products: synergistic effect of square motif and defective sites

If CO_2_RR stops at CO for Au and Ag catalysts, Cu presents the remarkable singularity to continue the electroreduction towards C_2_ products, hence involving a C-C coupling step. Experimental evidence shows that on the Cu surface, the electroreduction of CO_2_ to C_2_ products is pH-independent in the SHE scale^40^, suggesting that the rate-determining step (RDS) should not involve proton transfer. Considering that adsorbed CO (*CO) is the most abundant species from both experimental vibrational spectra^41^ and theoretical microkinetic models^42^, the dimerization of two *CO to form *OCCO has emerged as the most plausible C-C coupling mechanism, as supported by numerous studies^12,43,44^, although the existence of alternative coupling forms under specific conditions is acknowledged. To investigate the potential-dependent C-C coupling barriers, we performed constant potential transition state searches in the frame of GCDFT, as the *OCCO intermediate, characterized by a large dipole moment, is highly affected by the potential.

**Fig. 4:**
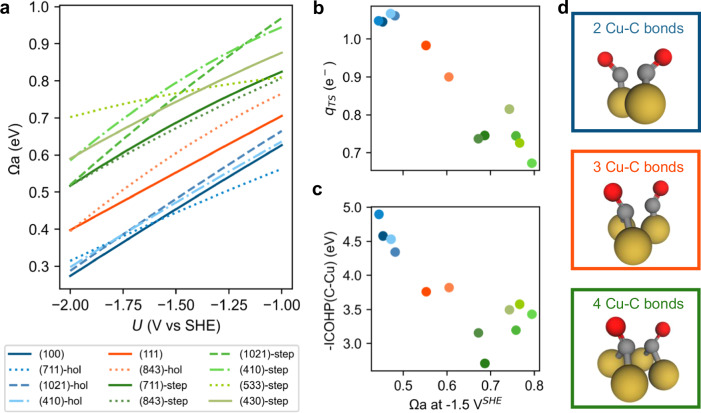
C-C coupling on Cu sites with different local structures. **a** C-C coupling barrier Ωa as a function of electrode potential on various Cu surfaces. **b** Electronic charge on the *OC-CO fragment in the TS (**c**) bond strength (negative of the integrated COHP, -ICOHP) between C and Cu for the C-C coupling TS as a function of the reaction barriers. **d** Schematic structure of the TS on three classes of sites, forming 2 Cu-C bonds, 3 Cu-C bonds, and 4 Cu-C bonds, respectively.

We explored the CO-CO coupling on different local sites of various surfaces based on the optimal CO coverages and configurations from the GCBH sampling at *P*_CO_ of 0.05 atm. For Cu(111) and Cu(100) surfaces, we considered two *top-*site adsorbed CO on the surface for C-C coupling. In the case of the stepped surfaces, two local sites are considered: pure step sites and *hollow* sites near the step. Our calculations reveal the structure-sensitive nature of C-C coupling, strongly dependent on the local arrangement of the Cu atoms, as depicted in Fig. 4a, illustrating the potential-dependent C-C coupling barriers on different sites. We classified these sites into three groups: square/quasi-square sites (four Cu atoms in a square arrangement, blue series), triangle sites (three Cu atoms in a triangle, red series), and step/kink sites (two Cu atom neighbors at the step edge/kink, green series). Square/quasi-square sites exhibit the highest activity for C-C coupling, followed by triangle sites, while step/kink dimer sites displayed the highest barriers. At − 1.5 V vs SHE, all the square/quasi-square sites demonstrate barriers around 0.45 eV, whereas the triangle sites show a barrier range from 0.55 eV to 0.6 eV. Notably, all the step/kink sites display barriers exceeding 0.65 eV (Fig. 4a and Supplementary Fig. 11).

Remarkably, Cu(111) has been previously regarded as the least active surface for C-C coupling. However, our calculations demonstrate that the coupling barrier on Cu(111) is not as unsatisfactory as previously reported^43,45^. This discrepancy arises from the fact that most calculations erroneously placed CO on *hollow* sites based on the site preference predictions from GGA functionals, leading to an unphysical, overestimated binding energy of *hollow*-site CO and thus positioning the initial state at a lower energy level. Moreover, it is commonly accepted that step sites facilitate C-C coupling, yet our results reveal that the intrinsic barriers on pure steps/kinks are too high to account for C_2_ activity^42,46^. One should underline that square/triangular sites can contain step site atoms so that low barrier paths can also exist on step/kink surfaces, but those active sites are not purely formed out of step edge atoms. A more detailed analysis of the activity of the various surfaces for C-C coupling will be discussed below.

Electronic analyses were performed on the transition state *OC-CO to understand the origin of such structure-sensitive C-C coupling barriers. Notably, a negative linear relationship can be observed between the negative charge accumulation on *OC-CO and the reaction barrier. On square/quasi-square sites, the *OC-CO fragment in the TS structures accumulates more than 1 *e*^-^, thus stabilizing the TS. In contrast, on triangle sites, the transition state shows a lower electron accumulation, while step/kink sites exhibited the poorest ability to donate electrons to the TS at the given potential of − 1.5 V vs SHE (Fig. 4b). A Crystal Orbital Hamilton Population (COHP) analysis^47^ was employed to quantify the bonding strength. Consistent with the charge analysis, we observed a relationship between the C-Cu bond strength for the C-C coupling transition state *OC-CO, expressed as the negative integrated COHP, and the reaction barrier (Fig. 4c). This observation can be rationalized by considering that square/quasi-square sites furnish four C-Cu bonds for the transition state while triangle-shaped sites and step/kink sites only supply three and two C-Cu bonds for TS, respectively, which results in a much stronger C-Cu bond strength observed on square/quasi-square sites (Fig. 4d). In short, the distinctive square termination of Cu atoms in square/quasi-square sites plays a crucial role in stabilizing the transition state *OC-CO by donating more electrons under the reductive potentials and providing a suitable spatial arrangement to accommodate *OC-CO, effectively stabilizing it by favorable C-Cu bond formation.

Although the potential-dependent barrier calculations highlighted the structure sensitivity of C-C coupling, they alone are insufficient to explain the observed reaction rate in CO_2_RR towards C_2_ products. Experimental studies have shown that planar Cu(111) and Cu(100) surfaces exhibit minimal CO_2_RR activity, contrary to what the barrier calculations suggest in terms of their ability for C-C coupling. This disparity underscores the importance of additional factors that influence the overall reaction rate in CO_2_RR. To better understand the trend of CO_2_RR to C2 products, a simple kinetic model was developed in this study. This approach, based on an analytical approximation, allows for the evaluation of reaction rates on different surfaces. We deliberately do not perform microkinetic analysis for two reasons. Firstly, the detailed reaction pathway from CO_2_ to C_2_H_4_ or C_2_H_5_OH involves 12e^-^ transfer, leading to numerous possibilities for potential reaction intermediates. In addition, the significant uncertainty associated with the first-principles modeling of proton-electron transfer barriers limits the applicability of microkinetic models in the context of electrocatalysis, specifically when the potential of energy surface (PES) is flat.

2*CO coupling is considered as the RDS, and the overall reaction rate is determined by the RDS. We account for different regions (*i*) on stepped and kinked surfaces, which include both the step/kink region and the remaining planar regions. The overall reaction rate is the sum of contributions from these different regions. According to the transition state theory, the reaction rate can be expressed as:

1 $${r}_{C2+}={\sum}_{i}\left(\frac{{k}_{B}T}{h}{\left[{\theta }_{{CO}}\right]}_{i}^{2}exp \left(-\frac{{G}_{a,i}(U)}{{k}_{B}T}\right)\right)$$

where ${\theta }_{{CO}}$ is the optimal CO coverage determined by GCBH and GCDFT, which corresponds to *P*_CO_ of 0.01 atm from the experiment. ${G}_{a}$ corresponds to the potential-dependent C-C coupling barriers, ${k}_{B}$ and ${h}$ represent Boltzmann’s constant and Planck’s constant, respectively. *T* is the temperature, which is set as 298 K.

For Cu(111) and Cu(100), the optimal CO coverage in steady state is predicted as 0 from GCBH and GCDFT. Considering the uniform surface sites and relatively weak CO binding on Cu(111) and Cu(100), for kinetics analysis, we applied the Langmuir adsorption model^48^ to determine the CO coverage of Cu(111) and Cu(100):

2 $$[{\theta }_{CO,i}]\,=\,({K}_{CO,i}{P}_{CO,i})/(1+{K}_{CO,i}{P}_{CO,i})$$

where the *P*_CO,i_ and *K*_CO,i_ represent the CO pressure and equilibrium constant for CO adsorption, *K*_CO,i_ is calculated as:

3 $${K}_{CO,i}=exp(-(\Delta {G}_{ads,i}(U))/({k}_{B}T))$$

Thus, the reaction rate on planar (111) and (100) surfaces can be expressed as:

4 $${r}_{c2+}=\frac{{k}_{B}T}{h}\,{\left[\frac{exp (-(\Delta {G}_{{ads},i}\left(U\right))/({k}_{B}T)){P}_{{CO},i}}{1+exp (-(\Delta {G}_{{ads},i}\left(U\right))/({k}_{B}T)){P}_{{CO},i}}\right]}_{i}^{2}exp \left(-\frac{{G}_{a,i}\left(U\right)+\Delta {G}_{{ads},i}(U)}{{k}_{B}T}\right)$$

**Fig. 5:**
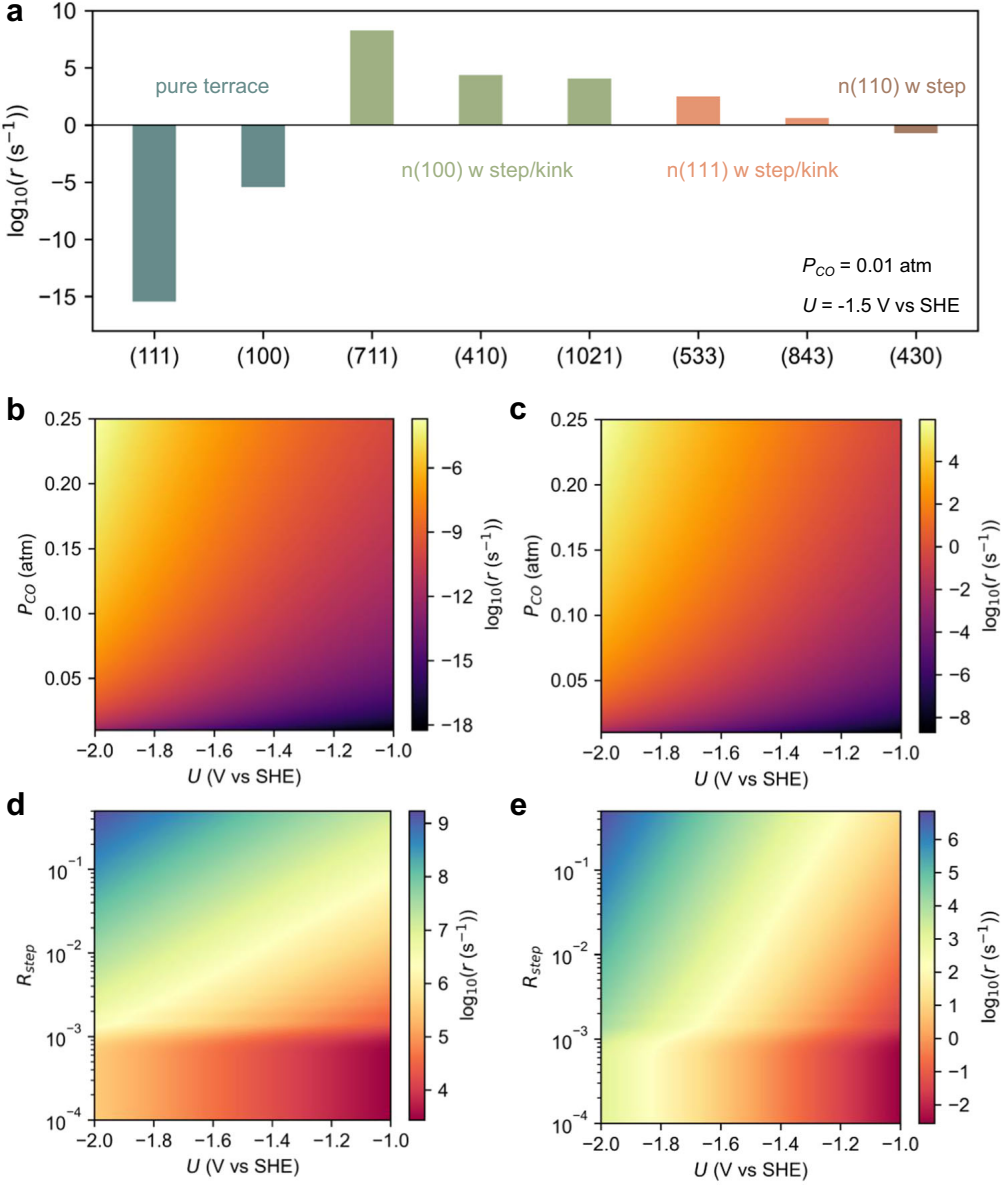
Model kinetic studies of the C2 product formation activity on various surfaces. **a** Calculated reaction rate at − 1.5 V vs SHE on various surfaces. Reaction rate as a function of potential and CO pressure on (**b**) Cu(111) and (**c**) Cu(100). Reaction rate as a function of potential and ratio of step (*R*_step_) on (**d**) Cu(S)-[n(100)*(111)] and (**e**) Cu(S)-[n(100)*(110)].

The kinetic analysis provides clear evidence that at -1.5 V vs SHE, *P*_CO_ of 0.01 atm condition, the CO_2_RR activity for generating C2 products on planar Cu(111) and Cu(100) surfaces is negligible (Fig. 5a and Supplementary Fig. 12). We observed that the Langmuir adsorption model predicts an extremely low CO coverage on Cu(111) and Cu(100) surfaces at low CO partial pressure (*P*_CO_ = 0.01 atm), with the value of 1.9 × 10^−^^6^ and 2.3 × 10^−^^4 ^ML, respectively, at the given condition (Supplementary Figs. 13 and 14). The low CO partial pressure originates from the sluggish CO_2_ conversion on planar surfaces, as mentioned before. The kinetic modeling findings are consistent with the experimental observations that clean planar Cu surfaces exhibit almost no CO_2_RR activity.

However, a significant increase in activity is observed when steps or kinks are introduced to the Cu(100) terrace, i.e., on surfaces such as Cu(711), Cu(410), and Cu(1021), all of which combine (100) terraces and step edges (Fig. 5a). Although these surfaces exhibit similar C-C coupling barriers compared to planar Cu(100), the substantial increase in CO coverage rationalizes the enhanced activity, as the reaction rate is proportional to the second order of the CO coverage. Similarly, the introduction of step or kink sites to the Cu(111) terrace leads to a marked increase in activity due to a significant rise in CO coverage, although these sites cannot match the activity of Cu(100) terrace with steps/kink (Fig. 5a). On the other hand, despite the high CO coverage observed on the Cu(110) surface, its inactivity in generating C2 products can be attributed to the highest C-C coupling barriers among the surfaces considered.

In general, the limited activity of pure planar Cu(111) and Cu(100) surfaces can be attributed to their low CO coverage, whereas Cu(111)/Cu(110) terraces with steps/kinks are limited by the high C-C coupling barriers. In contrast, Cu(100) terraces with steps/kinks exhibit superior activity for C2 products, as they feature both low C-C coupling barriers and high CO coverages simultaneously. Therefore, achieving high activity requires a synergistic combination of the square motif and step/kink sites. Specifically, the square motifs facilitate C-C coupling, while the undercoordinated sites promote high CO coverage. It is crucial to emphasize that relying solely on calculations of C-C coupling barriers, as commonly performed in many studies, is insufficient for accurately predicting the CO_2_RR activity towards C_2_ products on a specific catalyst.

As mentioned earlier, planar Cu(111) and Cu(100) surfaces are limited by their low CO pressure and coverage. However, increasing the CO pressure has the potential to enhance the C_2_ activity since a sharp increase in CO coverage can be observed (Fig. 5b, c). Direct CO reduction (COR) may address such a problem since it circumvents the sluggish CO_2_ conversion process on planar surfaces. Nevertheless, the high CO coverage induced by CO pressure tends to drive the restructuring of planar Cu surfaces towards step/kink surfaces, at least as predicted by thermodynamics. Furthermore, we show that when Cu(100) terraces are decorated by steps/kinks, the majority of the activity is contributed by the step/kink regions, while the other terrace regions still suffer from the unfavorable CO adsorption, as depicted in Fig. 5d, e. Increasing the density of (111) and (110) steps on Cu(100) terraces leads to a significant improvement in C_2_ activity, suggesting that enhancing the density of steps is an effective approach for enhancing CO_2_RR activity towards C_2_ products.

### The effect of H co-adsorption on CO_2_RR rate

Under reductive conditions, Cu surfaces will inevitably be partly covered with H, which will compete with CO adsorption and further influence CO_2_RR activity. We examined Cu(100) and Cu(711) surfaces as representative planar and stepped surfaces to elucidate the effect of H co-adsorption on CO coverage, C-C coupling, and reaction activity. Previous simulation efforts have shown that H coverage will not exceed 0.4 ML even under highly reductive potentials^42,49^. For our study, we assume an upper bound coverage of 4/9 ML on both Cu(100) and Cu(711) surfaces to explore the effect of H co-adsorption on CO_2_RR kinetics.

**Fig. 6:**
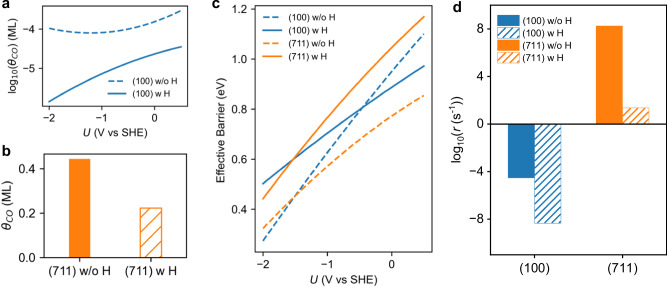
Effect of H co-adsorption on CO_2_RR on Cu surfaces. **a** CO coverage as a function of electrode potential on clean Cu(100) and H-covered Cu(100) surfaces. **b** CO coverage on clean Cu(711) and H-covered Cu(711) surfaces. The coverage does not change for a potential from − 2.0 V to 0.5 V vs SHE. **c** The comparison of effective barriers as a function of potential on clean and H-covered Cu(100) and Cu(711) surfaces. **d** The comparison of calculated reaction rates for CO_2_RR to C2 products on clean and H-covered Cu(100) and Cu(711) surfaces at − 1.5 V vs SHE.

On the Cu(100) surface, we found that at a CO pressure of 0.05 atm, CO adsorption is much more challenging compared to the clean Cu(100) surface, especially at negative potentials, resulting in a significantly lower estimated CO coverage (Supplementary Fig. 15 and Fig. 6a). On the Cu(711) surface, while the CO coverage on step edges remains unchanged, the coverage on near-terrace sites decreases due to competition with H adsorption, reducing the total CO coverage from 4/9 ML on the clean Cu(711) surface to 2/9 ML on the H co-adsorbed Cu(711) surface (Fig. 6b). The calculated effective C-C coupling barriers on both H-covered Cu(100) and Cu(711) show an increase at negative potentials, compared to that on clean surfaces (Fig. 6c).

We then conducted kinetic analysis on H-covered Cu(100) and Cu(711) surfaces to elucidate the effect of H co-adsorption on CO_2_RR activity (Fig. 6d). On the Cu(100) surface, despite no significant increase in the reaction barrier, the predicted reaction rate is much lower at negative potentials compared to the clean Cu(100) surface due to the lower CO coverage. On the H-covered Cu(711) surface, the decreased reaction rate mainly results from the increased C-C coupling barrier, with CO coverage on step regions remaining relatively unchanged. However, the trend remains that the reaction rate for CO_2_RR on H-covered Cu(711) is much higher than on Cu(100) surface, the latter showing almost no activity for CO_2_RR.

Overall, H co-adsorption alters the absolute reaction rates on both planar and stepped surfaces, but the trend and limiting factors remain the same. On the planar Cu(100) surface, CO adsorption becomes more challenging with H co-adsorption, maintaining ultralow CO coverage as the limiting factor, which has also been indicated by our recent work^50^. On stepped surfaces, strong CO bonding at step edges enables CO coverage to remain stable, though the increased C-C coupling barrier decreases the overall reaction rate. Modeling H and CO co-adsorption provides more realistic results, yet the mechanisms and conclusions drawn without H co-adsorption remain unchanged.

### Experimental evidence on well-defined single crystal Cu surfaces

**Fig. 7:**
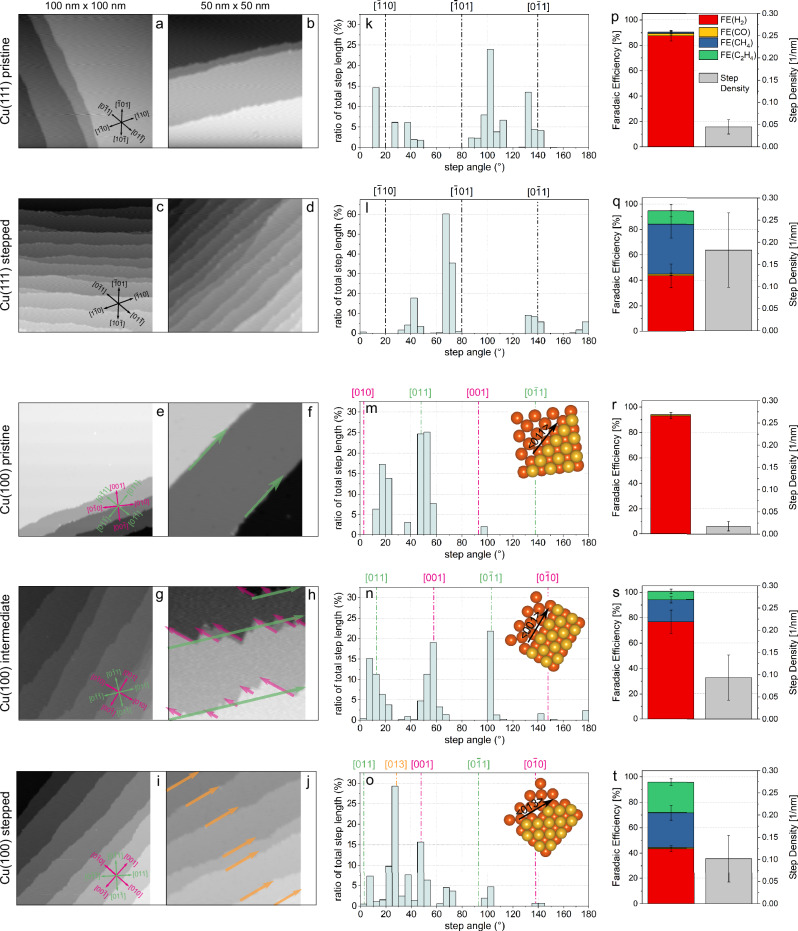
Impact of surface morphology on CO_2_RR selectivity for well-defined UHV-prepared Cu(111) and Cu(100) single crystals. LT-STM images on UHV-prepared (**a**, **b**) pristine Cu(111); **c**, **d** Cu(111) surface after five successive cycles of CO_2_RR followed by sputtering and subsequent annealing in UHV (noted as stepped Cu(111)); **e**, **f** pristine Cu(100); **g**, **h** intermediate Cu(100) and (**i**, **j**) stepped Cu(100), which are Cu(100) surface after one and two CO_2_RR cycles, followed by sputtering and subsequent annealing in UHV, respectively. The distribution of the step edge length in dependence on the step edge angle of the respective surfaces is shown in (**k**–**o**). For Cu(100), the insets show an atomic model of the most prominent step-edge direction. The Faradaic Efficiencies (FE), which are obtained after 1 h of CO_2_RR at − 1.1 V vs. RHE in 0.1 M KHCO_3_, as well as the calculated step edge density for the respective surfaces, are displayed in (**p**–**t**). The LT-STM image in (**d**) and FE data are partially adapted from Nguyen et. al with permission^10^. For electrochemical measurements, the error bars stem from averaging the product selectivities over 1 hr. For step density, the error bar stems from the standard variation of the measured step edges.

The theoretical predicted mechanisms are further validated by experimental evidence on well-defined single-crystal Cu surfaces. Scholten et al. have shown that only by introducing defects and high index sites with harsh treatments, such as chemical etching, one can achieve on well-defined Cu single crystal surfaces product distributions typically reported in the previous literature for electropolished single crystals^1,9^. Recent work on UHV-prepared Cu(111) surfaces has unveiled that highly relevant defects for hydrocarbon production are step edges^10^. Figure 7 illustrates the impact of reaction history and associated surface morphology for well-defined Cu single-crystal surfaces on CO_2_RR selectivity. Figure 7a–j shows low-temperature scanning tunneling microscopy (LT-STM) images of atomically flat ultra-high vacuum (UHV)-prepared Cu(111) and Cu(100) single crystal surfaces. The pristine Cu(111) surface is shown in Fig. 7a, b, and the same surface after five successive cycles of CO_2_RR, followed by sputtering and subsequent annealing in UHV, are shown in Fig. 7c,d. For Cu(100), the pristine surface is shown in Fig. 7e, f, the intermediate surface in Fig. 7g, h after one, and the “stepped” surface in Fig. 7i, j after two CO_2_RR and sputter/anneal cycles. Details of the methods and experimental conditions can be found in previous work by some of the co-authors^9,10^. Figure 7k–o shows the distribution of step edge length in dependence on the step angle, which was measured between the step edges and the horizontal axis in the STM images. For each surface, the crystallographic directions were determined based on atomically resolved STM images or Low Energy Electron Diffraction (LEED) and put in relation to the measured step angle. Figure 7p–t displays the product distribution for CO_2_RR as well as the step density obtained from the STM images (see Supplementary Fig. 19 and 20) on the respective surfaces. For Cu(111), the step density is increased by a factor of ~ 4.1 from the pristine to the post CO_2_RR surface, whereas for Cu(100), the step density is increased by a factor of ~ 5.2 between the pristine and intermediate surface, whereas the intermediate and stepped surface have almost identical step density. Note that cycles of sputtering and annealing in UHV without CO_2_RR periods do not produce the restructuring and step density increase, but an atomically flat surface. In addition, the different product distributions depend on the preparation and utilization history of the single crystal. The pristine planar Cu(111) and Cu(100) surfaces only produce hydrogen in CO_2_RR, whereas the stepped post-reaction surfaces produce hydrocarbons. The change in surface morphology from a pristine towards a stepped surface, despite re-generative UHV preparation steps in between, suggests major irreversible restructuring processes taking place during CO_2_RR. These experimental findings support our theoretical insight that the pristine, atomically flat surfaces tend to restructure in reaction conditions to the active stepped surfaces. As shown by our calculations, the stepped surfaces are stabilized by the CO coverage in the CO_2_RR reaction condition, and therefore, further steps tend to be formed during the reaction, supporting the drastic morphological evolution from the pristine to the ‘CO_2_-used and UHV-regenerated’ stepped surface. For Cu(100), the steps on the pristine surface tend to align along the crystallographic <011> direction (green arrows), and on the intermediate Cu(100), a reorientation along the <001> direction (pink arrows) is observed. This is well in line with the calculations showing that bare Cu(711) (with steps along <011>) is markedly more stable than Cu(410) (with steps along <001>) while under a coverage of CO Cu(410) is strongly stabilized versus Cu(711) (see Fig. 2). A similar step edge reconstruction under electrochemical conditions caused by CO adsorption and desorption was observed via operando electrochemical STM elsewhere^51^. Furthermore, on the stepped Cu(100) surface, an additional preferred direction <031> (yellow arrows) can be observed, which is a kinked step edge. This aligns with our calculations as well, which indicate that CO adsorption leads to significant stabilization on the Cu(1021) surface (with step along <031>), which is otherwise highly unstable without CO adsorption (see Fig. 2). Moreover, it is also plausible that oxygen species might be dissolved into our Cu single crystals during the successive CO_2_RR cycles, and that the presence of such species, in quantities too small to be detected experimentally, might help to stabilize the new steps observed. We also learn from Fig. 7 that the CO_2_RR activity is predominantly governed by defect structures such as step edges. The HER is favored on both pristine, atomically flat Cu(111) and Cu(100) surfaces, which backs our explanation that pristine flat (111) and (100) surfaces suffer from extremely low CO coverage and unfavorable CO binding, which limits their CO_2_RR activity. Only by increasing the step density on these surfaces the selectivity for hydrocarbon production can be increased. In addition, not only the step density but also the orientation of the step edges plays a crucial role. Although the step density is almost identical for the intermediate and stepped Cu(100) surface, the alignment of the step edges along <001> and the additional formation of kinked step edges results in a doubling of hydrocarbon products. On both stepped Cu(100) and Cu(111) surfaces, the selectivity for hydrocarbon increased from zero to ~ 50% Faradaic Efficiency (FE). This observation supports our theoretical insights that steps and kink sites enable a beneficial CO_2_ activation and higher CO coverage, leading to a significant increase in CO_2_RR activity. As soon as the CO coverage is increased on the surface by additional steps and kink sites, the rate of C-C coupling is determined by intrinsic facet effects. Although the amount of produced hydrocarbons is almost identical for both Cu(111) and Cu(100), the product selectivity differs greatly regarding the exact hydrocarbon product, which unveils the key role of the initial surface structure of the pre-catalyst surface, since it determines it subsequent operando transformation. Cu(100) shows a much higher selectivity towards ethylene than Cu(111), which favors the methane production. This finding is in agreement with our theoretical calculations, which have shown that a ‘square’ Cu ensemble in near vicinity to step edges are the active sites for C-C coupling, leading to a higher hydrocarbon formation rate for (100) surfaces with steps and kinks as seen in Fig. 5a. Thus, both experiments and theoretical modeling suggest that defects on Cu, that are also formed during CO_2_RR, enable the reduction of CO_2_ into CO and adjacent square-motif Cu enables the C-C coupling. However, the detailed local atomic structures would need to be identified from additional experiments and modeling in future work.

## Discussion

In summary, we provide from first principles calculations a comprehensive analysis of the structure sensitivity for CO_2_RR on Cu catalyst and of the tendency for various surface terminations to restructure under reactive conditions. The latter is also illustrated here based on experimental nanoscale microscopy data obtained from Cu(111) and Cu(100) surfaces. We demonstrate that a thorough understanding of the site preference and an accurate energetic description of CO adsorption is essential to present a consistent picture of CO-covered Cu terminations. We showed that on planar Cu(111) and Cu(100) surfaces, the initial CO_2_ activation is particularly challenging, due to a markedly endergonic CO_2_ chemisorption, which impedes CO generation. This finding is backed up by experimental data on pristine atomically flat UHV-prepared surfaces. This finding has been extended to other transition metals in which CO_2_ activation is decisive, such as Ag and Au. The further step of C-C coupling between CO adsorbates is a structure sensitive reaction, however, we emphasize that the sole coupling barrier is not a sufficient descriptor of the surface activity toward C_2_ products generation. Rather, the rate depends on both the CO coverage and the intrinsic coupling barrier. The kinetic analysis demonstrates that planar Cu(111) and Cu(100) exhibit minimal CO_2_RR activity for C_2_ products, and the majority of the observed activity arises from the step or kink region. The experimental data confirm that the increase in step density from pristine to stepped surfaces leads to the production of hydrocarbons on the latter. Our stability diagram shows that the planar Cu(111) and Cu(100) surfaces are the most stable in vacuum or under a reductive potential without CO adsorption, and stepped/kinked surfaces are less stable and less probable. However, in CO_2_RR conditions, upon CO adsorption and under negative potential, the relative stability of the Cu surfaces is inverted: stepped and kinked surfaces become more stable than planar low-index (111) and (100), due to the stronger CO adsorption on the former. This thermodynamic stability inversion is the driving force for the restructuring of Cu surfaces in CO_2_ electroreduction conditions, where low activity planar (111) and (100) surfaces would tend to facet into highly active stepped and kinked terminations.

These results suggest that the commonly used close-packed (111) and (100) surfaces of transition metals are not appropriate models for studying the mechanisms of CO_2_RR. Instead, our models demonstrate from first principles that the presence of step or kink sites on the catalyst surface is primarily responsible for the overall activity. This provides understanding and support to experimental findings on the role of defects, which are also created during the reaction in the CO_2_RR environment. Moreover, we propose that the most favorable local sites on Cu for CO_2_RR to C_2_ products are square motifs adorned with steps, kinks, or other low-coordinated sites, as they feature both the facile C-C coupling barriers and high CO coverage and show the synergistic effect towards overall reaction rate. Meanwhile, we emphasized the role of low-coordinated defective sites for CO_2_ activation. This is relevant to recent work, that there exist distinct sites for CO_2_-CO and CO conversion on Cu^52^. We suggest that defective Cu will be responsible for the reduction of CO_2_ into CO, while nearby square-motif Cu serves as active sites for C-C coupling.

In addition, our study indicates a strong thermodynamic driving force for the restructuring of planar surfaces to form step and kinked surfaces under the CO_2_ reactive conditions and associated CO adsorbate coverage. supports the concept of self-activation of inert planar surfaces through restructuring, although this requires mass transport of Cu atoms, which could be kinetically limited. For example, the restructuring of a “perfect” Cu(111) single crystal surface under CO_2_RR, although thermodynamically favorable, could be challenging since CO does not absorb in a stable manner at ambient pressures, so that a perfect (111) surface might be maintained in a metastable state for a long time. More realistic catalysts with a polycrystalline structure showing small (111) and (100) inactive facets upon preparation might readily restructure during reaction toward the formation of highly active (711) or (410) facets, for example^13,14^. Finally, several approaches for improving CO_2_RR activity can be proposed, including the synthesis of catalysts with abundant square-step sites, such Cu(S)-[n(100)×(111)] or Cu(S)-[n(100)×(110)], and the development of methods such as in situ potential pulses, to accelerate the surface restructuring under the reactive conditions.

## Methods

### Model Setup

Cu(111), Cu(100), Cu(711), Cu(430), Cu(843), Cu(1021), Cu(533) and Cu(410) were modeled by 4-layer (3 × 3), (3 × 3), (1  ×3), (1 × 3), (1 × 1), (1 × 1), (1 × 3) and (1 × 3) slabs, respectively. Note that Cu(1021) stands for Cu(10 2 1) facet. The bottom two layers of the slab were constrained as the bulk region, and everything else was allowed to relax as the interface region. A vacuum layer of 15 Å thickness was added in the *Z*-direction to avoid spurious interactions between periodic images.

### DFT Calculations

DFT calculations were performed using the Vienna Ab-Initio Simulation Package (VASP) with RPBE functional^53,54^. The core electrons were described with the projector augmented wave (PAW) method^55^. The cutoff energy for the kinetic energy of the plane waves was 450 eV. The entropy corrections from frequency calculations were computed using the harmonic oscillator approximation. The convergence criteria for electronic and force minimization were set to 10^−5^ eV and 0.02 eV/Å during the global optimization and 10^−6^ eV and 0.01 eV/Å for the final refinement. The Brillouin zone was sampled using the (3 × 3 × 1) Gamma-centered k-point grids during the global optimization, and (6 × 6 × 1) for further calculations and subsequent GCDFT. The transition states (TS) were located using the climbing image nudged elastic band (CI-NEB) method with image-dependent pair potential (IDPP) interpolation^56^. Each TS geometry was confirmed to have only one imaginary mode.

All electronic structure analyses were performed based on a converged charge density or wavefunction. The Bader charges were calculated using the Bader Charge Analysis program^57^. The COHP analysis was performed using the LOBSTER program with the pbeVaspFit2015 basis set^47^.

### Grand canonical basin hopping global optimization

The basin hopping algorithm is a type of Monte Carlo method combined with local minimization to convert the potential energy surface (PES)^21,22,58^. The Monte Carlo sampling is done through atomic displacements and the Metropolis criterion at room temperature. The displacements are done on the CO, whereas the Cu slab is allowed to relax in response but is not explicitly sampled (Supplementary Figs. 17 and 18). The free energy is calculated by subtracting the reference chemical potential (which is a function of temperature and pressure) of the adsorbate from the energy of the system as follows:

5 $$\Delta G=E({n}_{{CO}}+{slab})-E({slab})-{n}_{{CO}}\times {\mu }_{{CO}}$$

where *E*(*n*_CO_ + slab) is the electronic energy of the optimized structure, *E*(slab) is the energy of the optimized bare Cu slab, and *µ*_CO_ is the chemical potential of CO in the gas phase. Translational and rotational terms are taken into account to calculate the CO gas phase chemical potential, but vibrational terms are not included, since they are neglected in the CO adsorbed state as well. In this work, we choose the *T* = 298 K and *P*_CO_ = 0.05 atm, which corresponds to the CO partial pressure measured on polycrystalline Cu. Grand canonical global optimization methods, which approximate the near-equilibrium structures that would emerge over long timescales, provide a suitable framework for studying restructuring trends. Although molecule dynamics may provide more information on the kinetics, it is limited in capturing experimentally relevant restructuring timescales.

### Grand canonical density functional theory

Grand Canonical DFT (GCDFT) calculations are performed to obtain the potential-dependent energetics, where the number of electrons is allowed to change to adapt to the change of work function along the reaction pathway. The potential-dependent grand canonical free energy is expressed within the surface charging model as follows:

6 $${{\Omega }}\left(U\right)={{\Omega }}\left(U\right)-q(U)\cdot {FU}={{\Omega }}\left({U}_{0}\right)-\frac{1}{2}C{\left(U-{U}_{0}\right)}^{2}$$

where $\Omega \left(U\right)$ is the electronic energy of the surface at the *U* potential in the SHE scale, $q(U)$ is the charge difference against the neutral condition, *F* is the Faradaic constant. *C* stands for the effective capacitance, and *U*_o_ represents the potential of zero charge (*pzc*). *C* and *U*_0_ are obtained by fitting the quadratic relationship of the energies as a function of potential. For more details of GCDFT, see our previous work^24,27,59^. The self-consistent implicit solvation model VASPsol^60^ is used to represent the polarizable electrolyte. The dielectric constant of 78.3, and the Debye screening length 3 Å are used, corresponding to the bulk ion concentration of 1 M. The effects of the dielectric constant on the adsorption energy and physical properties of the electrochemical interface are discussed in Supplementary Note 4. The effect of field-dipole interaction on stability is discussed in Supplementary Note 2, and the cation effect is discussed in Supplementary Note 7.

### RPA calculations

We calculate total energies using the Adiabatic Connection Fluctuation Dissipation Theorem (ACFDT) in its Random Phase Approximation (RPA), a method originating from many-body perturbation theory that has been reformulated within the framework of density functional theory^30^.

### Single crystal preparation

Cu single crystals (MaTeck) were prepared in UHV via sputtering and annealing. The crystals are sputtered with Ar^+^-ions at a voltage of 1700 V and current of 12 µA for 30 min. Subsequently, the crystal is annealed at *T* = 650 °C for 10 min. These sputter and annealing cycles are repeated until a sharp Low Energy Electron Diffraction (LEED) pattern is obtained. Afterwards, four final preparation cycles are completed with milder sputter conditions using a voltage of 700 V and a current of ~ 8 µA, followed by annealing at *T* = 650 °C for 10 min.

### Electrolyses

After UHV preparation, CO_2_RR was measured ex-situ in air on single-crystal surfaces using a standard glass H-cell. The single crystals were mounted in a custom-built sample holder, as described in a separate reference^61^.

The electrochemical setup included a platinum mesh counter electrode (99.9% purity, MaTeck), a leak-free Ag/AgCl reference electrode (LF-2-100, Alvatek), and a Selemion membrane. A 0.1 M KHCO_3_ electrolyte (ACS reagent, 99.7%, Sigma-Aldrich, pH = 6.8) was used and bubbled with CO_2_ for 30 min prior to measurement.

In this work, the resistance (R) was determined before each electrolysis to verify the correct assembly of the electrochemical cell and sample holder, ensuring stable electrical contact. The reference electrode was calibrated against the Normal Hydrogen Electrode (NHE) in H_2_-saturated 0.1 M KHCO_3_. The measured resistance in the H-cell setup with the in-built sample holder ranged between 62.5 Ω ± 12.5 Ω.

During CO_2_RR, iR compensation was applied via the current-interrupt method. The cell volume was 25 mL per compartment. The flow rate was maintained at 20 mL/min and monitored throughout the reaction to ensure accurate calculation of the Faradaic efficiency. It was controlled and adjusted using a mass flow controller (Bronkhorst EL-Flow Select) and verified with a portable flow meter (Agilent ADM Flow Meter G6691A).

Gaseous products were detected every 15 minutes using a gas chromatograph. The error bars in the product distribution represent the standard deviations from four measurements taken over the course of one hour. Product analysis followed a similar procedure to that described in references^9,61^.

In this study, liquid product analysis was not considered, as the typical amounts detected were below 10% of the Faradaic efficiency. Focusing on gaseous products provides a more accurate assessment of the dominant reaction pathways and efficiency measurements.

## Supplementary information

Supplementary Information

Description of Additional Supplementary Files

Supplementary Data 1

Transparent Peer Review file

## Source data

Source Data

## Data Availability

The data supporting this study’s findings are available in the supporting information (supplementary Data 1) and Source Data file. Source data are provided in this paper.
