# Supplementary material for: Structure Sensitivity and Catalyst Restructuring for CO2 Electro-reduction on Copper
Source: Nat Commun. 2025 Apr 30;16:4064. doi: 10.1038/s41467-025-59267-3 (PMC12043938; doi:10.1038/s41467-025-59267-3)
Supplement: Supplementary file 1 — Supplementary Information [file 41467_2025_59267_MOESM1_ESM.pdf]

Supplementary Information for

Structure Sensitivity and Catalyst Restructuring for CO_2 Electro-reduction on Cu

Dongfang Cheng, Khanh-Ly C. Nguyen, Vaidish Sumaria, Ziyang Wei, Zisheng Zhang, Winston Gee, Yichen Li, Carlos G. Morales-Guio, Markus Heyde, Beatriz Roldan Cuenya, Anastassia N Alexandrova, Philippe Sautet**

* Correspondence: ana@chem.ucla.edu, sautet@ucla.edu

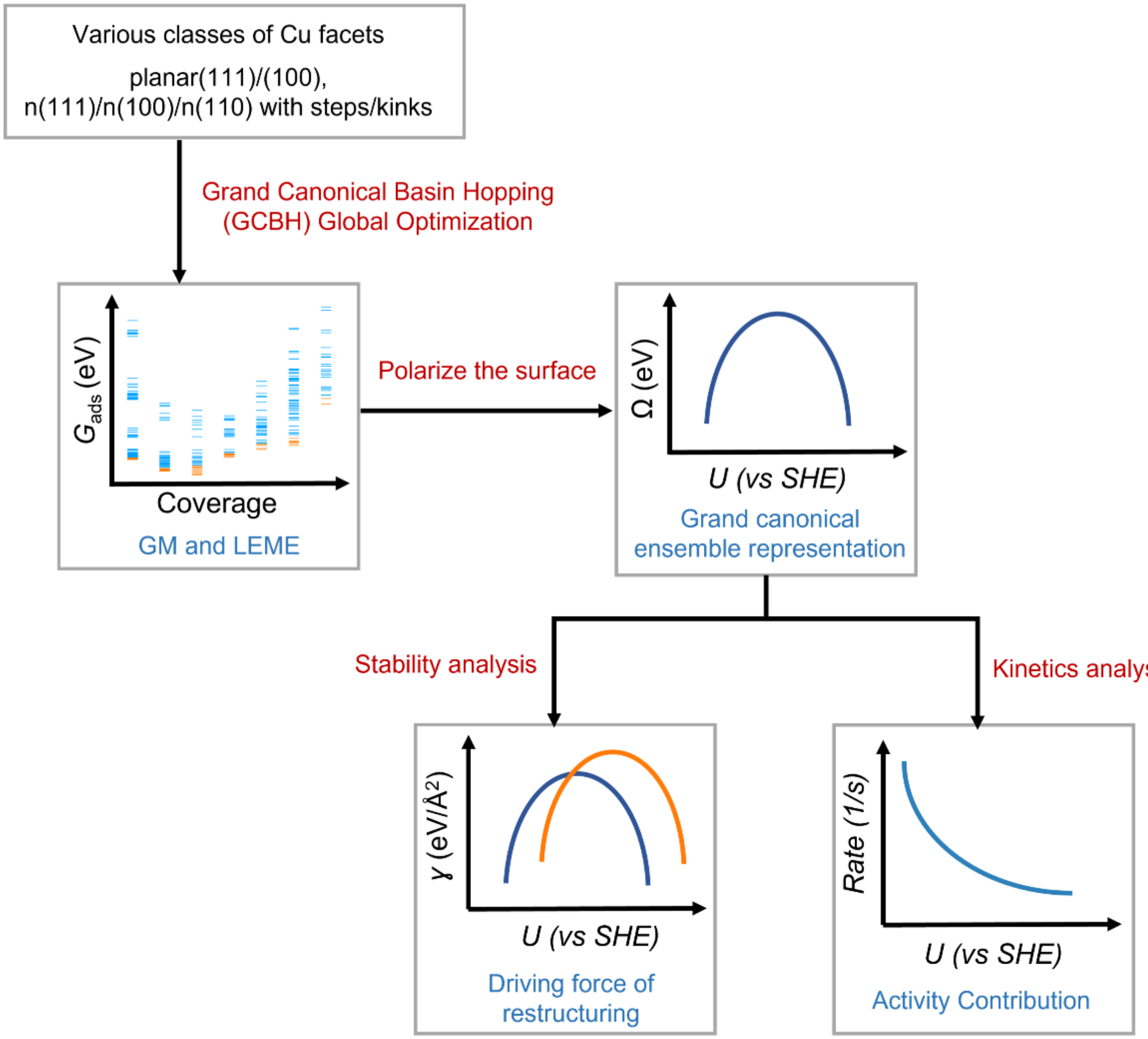

Supplementary Figure 1. Workflow for understanding structure sensitivity and restructuring of Cu in CO_2R. Combining grand canonical basin hopping global optimization and grand canonical DFT calculations to get the grand canonical ensemble representation in structure and energy. Stability analysis was performed to uncover the thermodynamic driving force for the restructuring while the kinetic model was developed to unveil the structural sensitivity in activity.

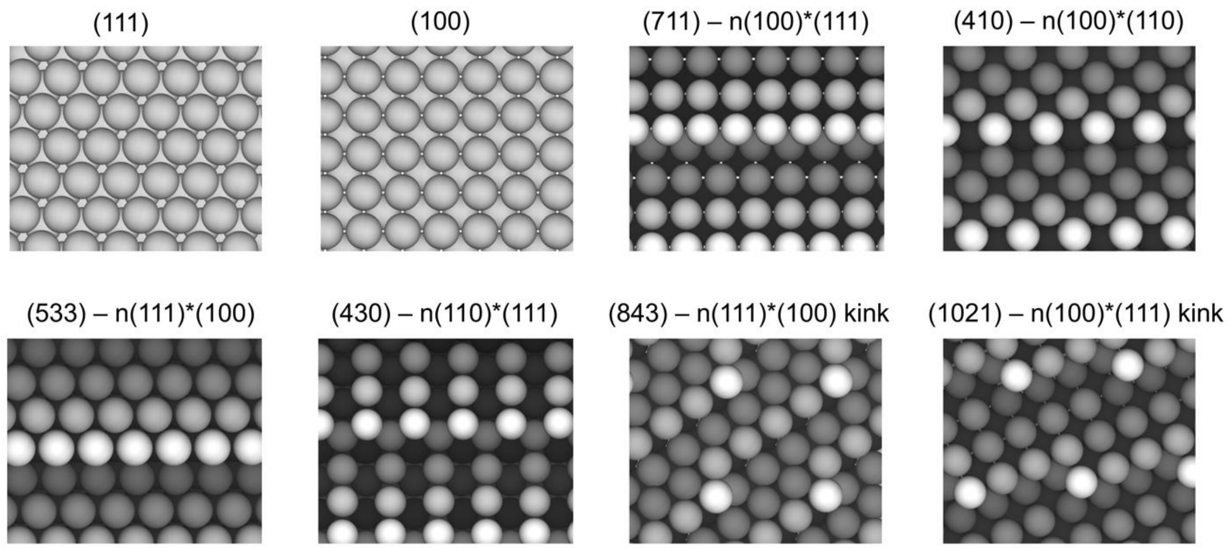

Supplementary Figure 2. Ball models of various clean surfaces considered. Colors represent the z-position of the atoms. White balls represent the step/kink edge and others represent the terrace.

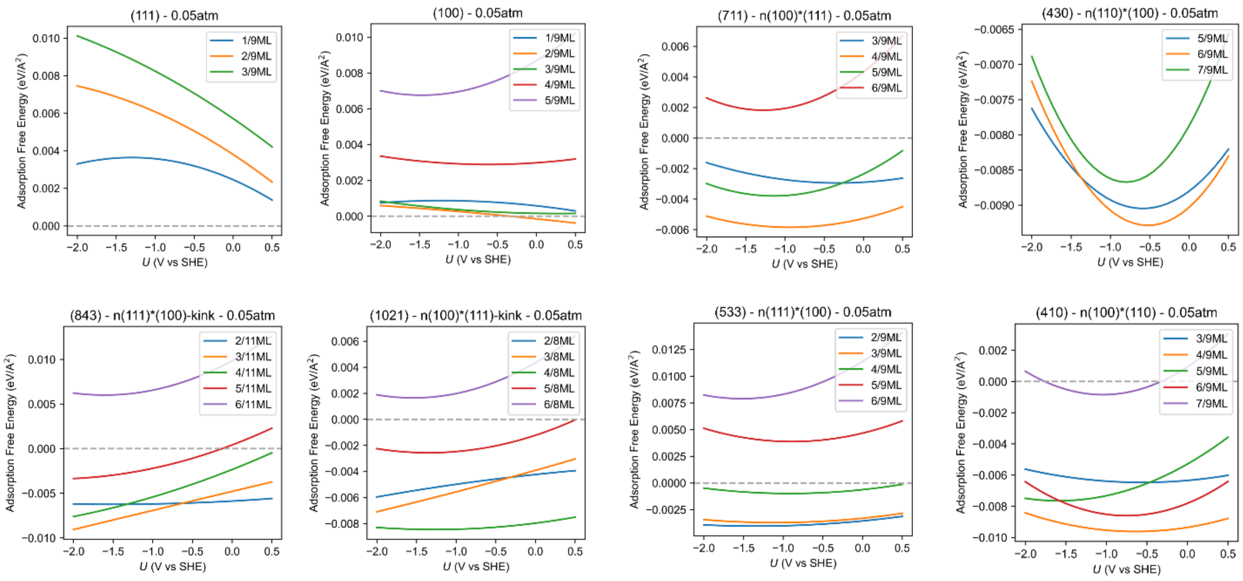

Supplementary Figure 3. Adsorption free energy of CO as the function of potential on various surfaces under the CO pressure of 0.05atm.

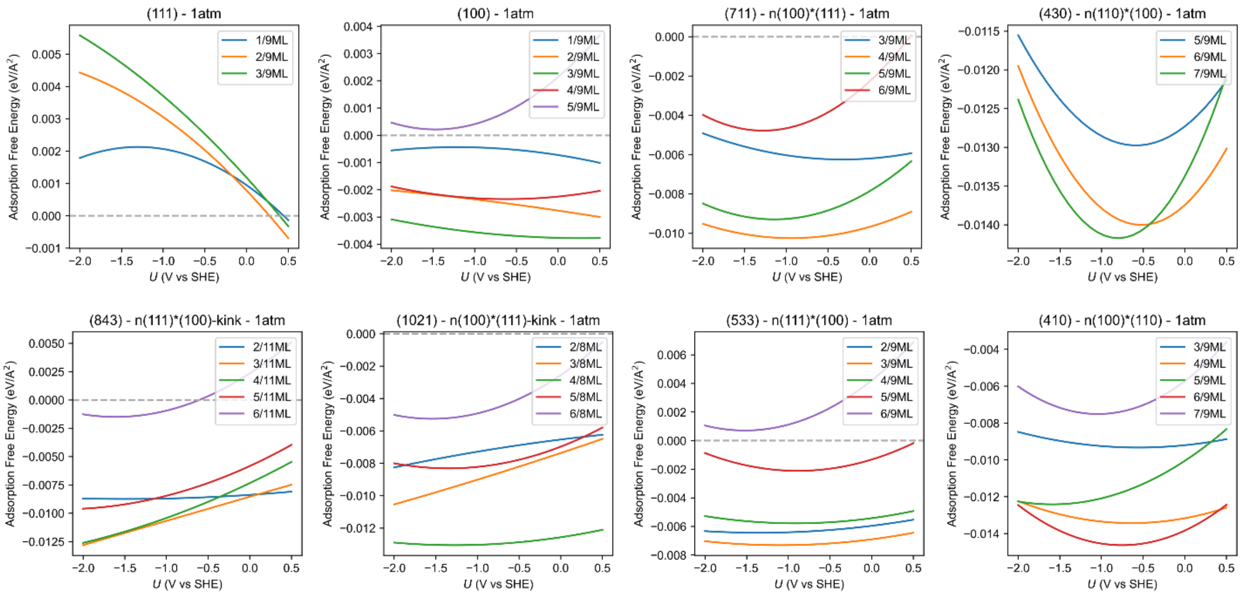

Supplementary Figure 4. Adsorption free energy of CO as the function of potential on various surfaces under the CO pressure of 1atm.

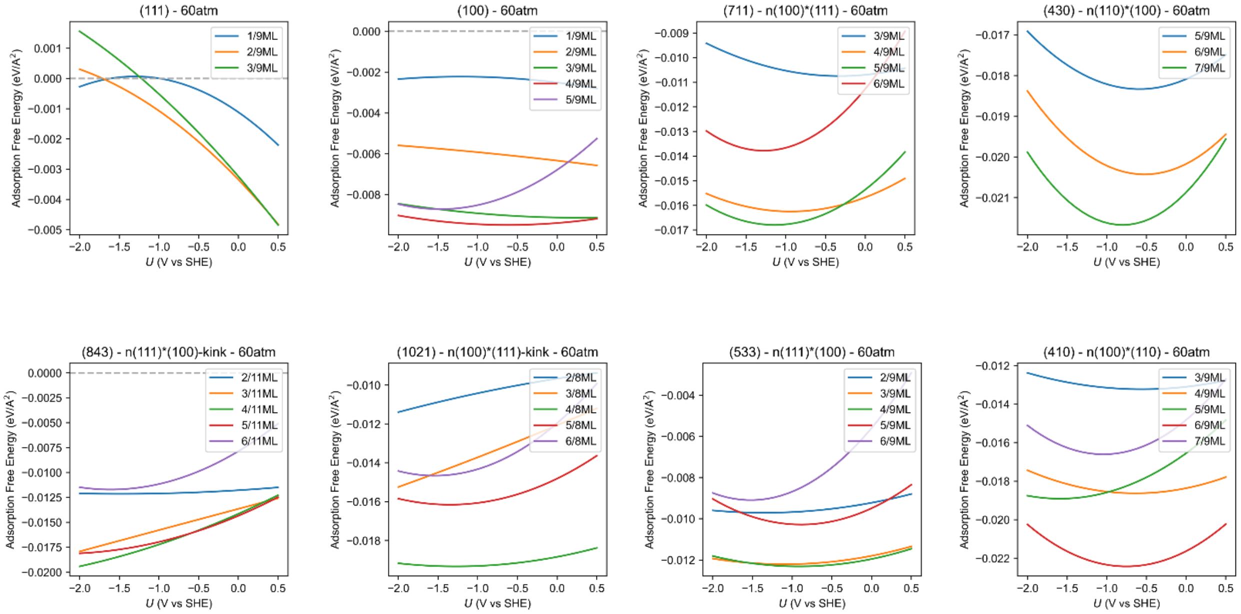

Supplementary Figure 5. Adsorption free energy of CO as the function of potential on various surfaces under the CO pressure of 60atm.

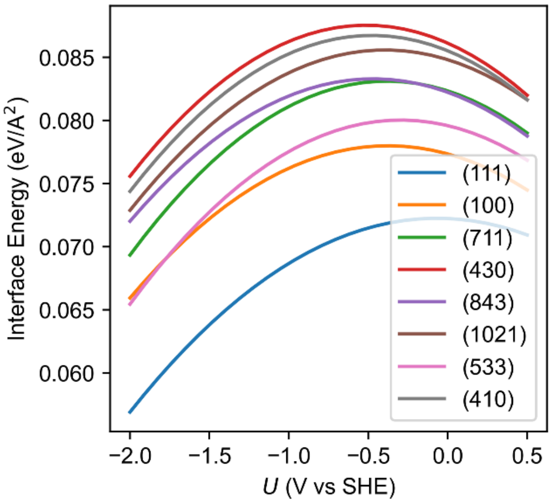

Supplementary Figure 6. Surface energy of various surfaces as the function of potential without CO adsorption.

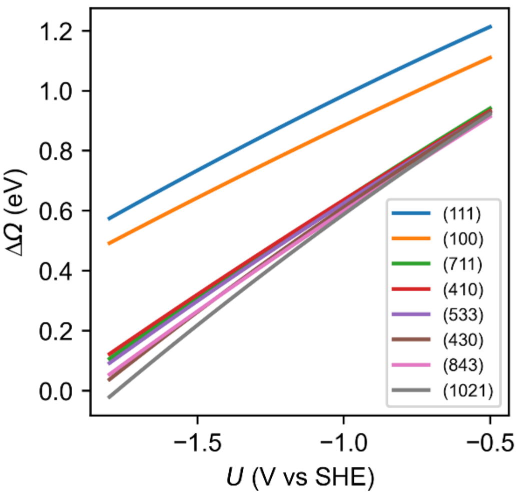

Supplementary Figure 7. Adsorption free energy of CO_2 as the function of potential on various Cu surfaces.

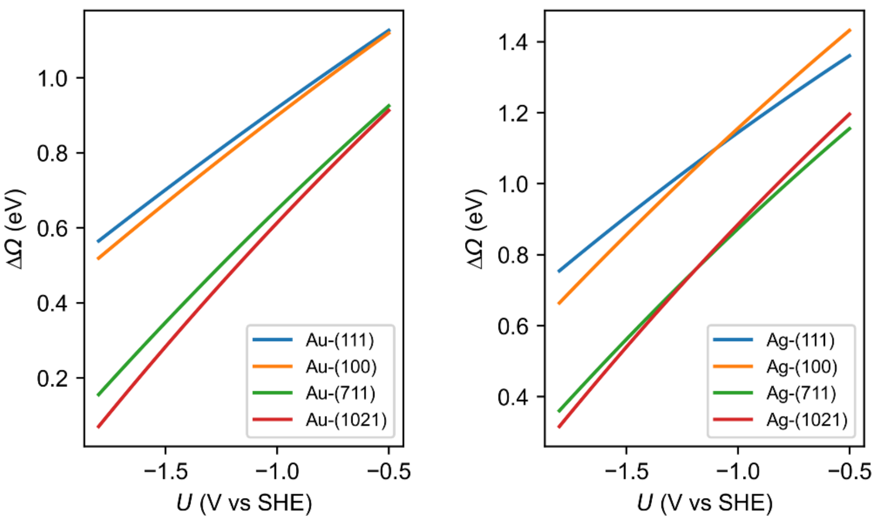

Supplementary Figure 8. Adsorption free energy of CO_2 as the function of potential on various Au and Ag surfaces.

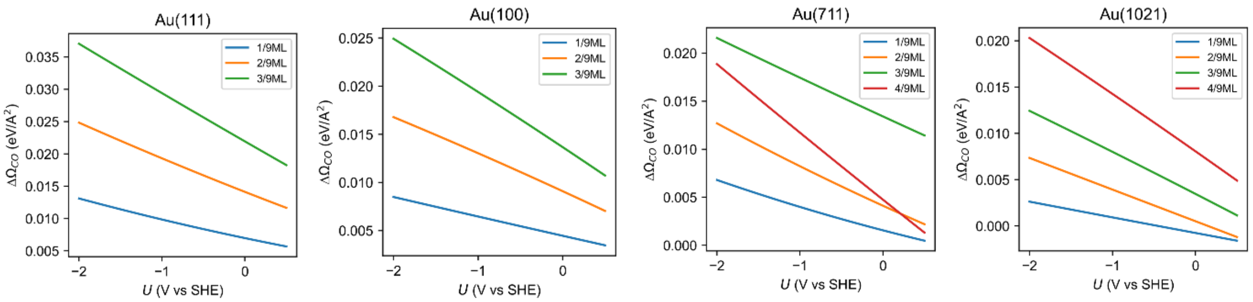

Supplementary Figure 9. Adsorption free energy of CO as the function of potential on various Au surfaces.

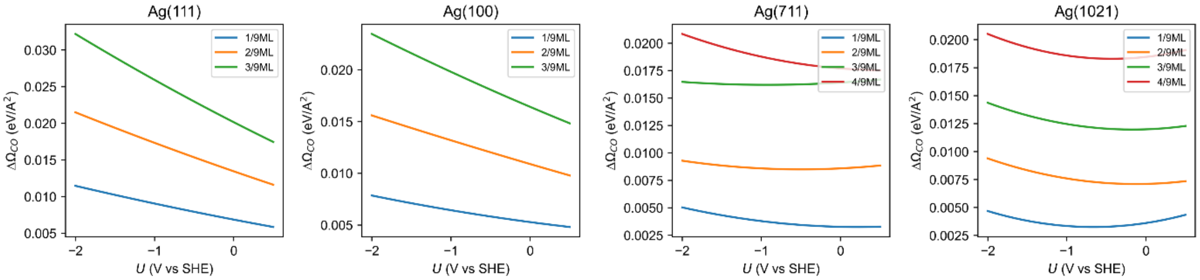

Supplementary Figure 10. Adsorption free energy of CO as the function of potential on various Ag surfaces.

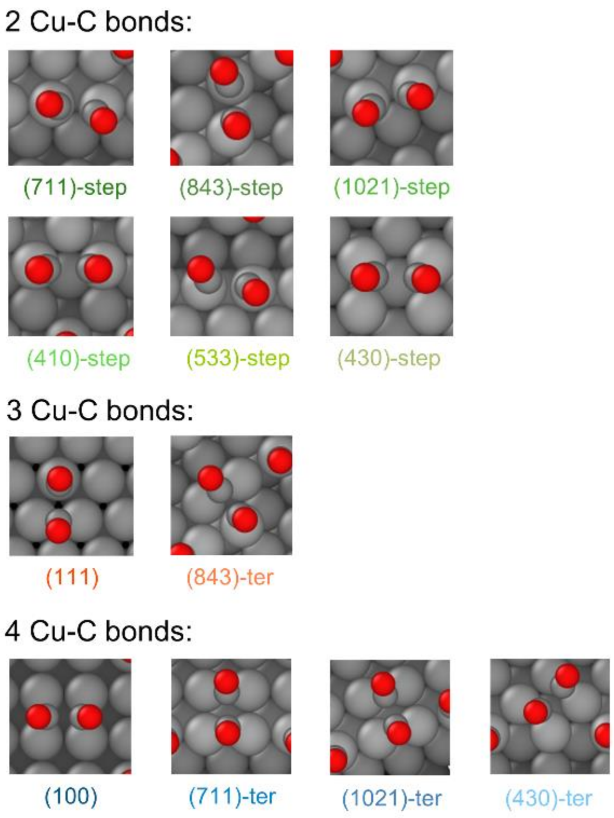

Supplementary Figure 11. Geometries of *OC-CO transition state on different local structures.

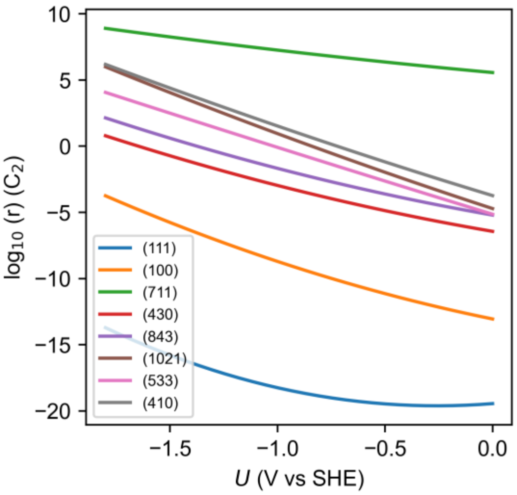

Supplementary Figure 12. The reaction rate of CO_2R to C2 products as the function of potential on various Cu surfaces.

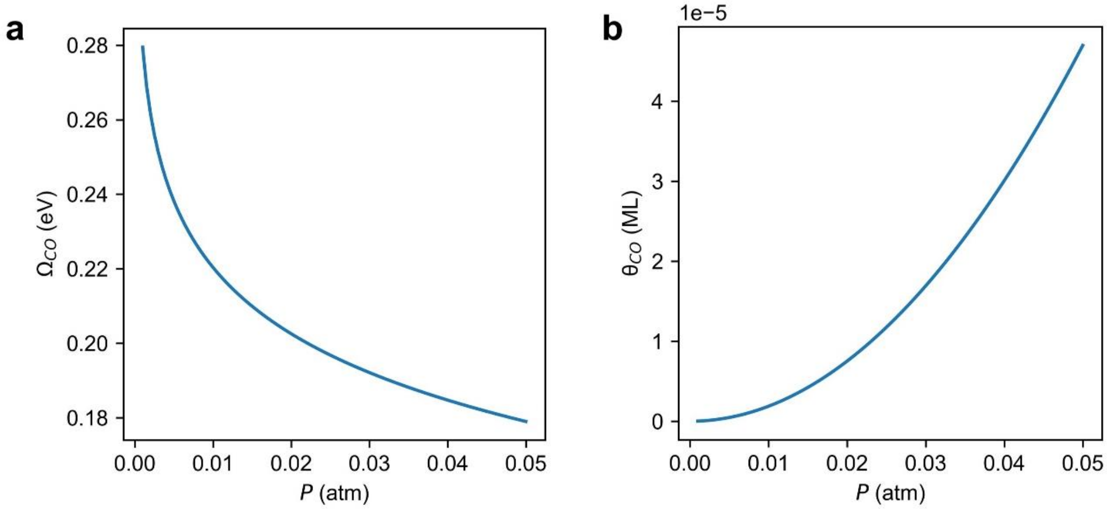

Supplementary Figure 13. (a) Adsorption free energy of CO as the function of CO pressure on Cu(111) at -1.5 V vs SHE. (b) CO coverage as the function of CO pressure on Cu(111) at -1.5 V vs SHE based on Langmuir adsorption model.

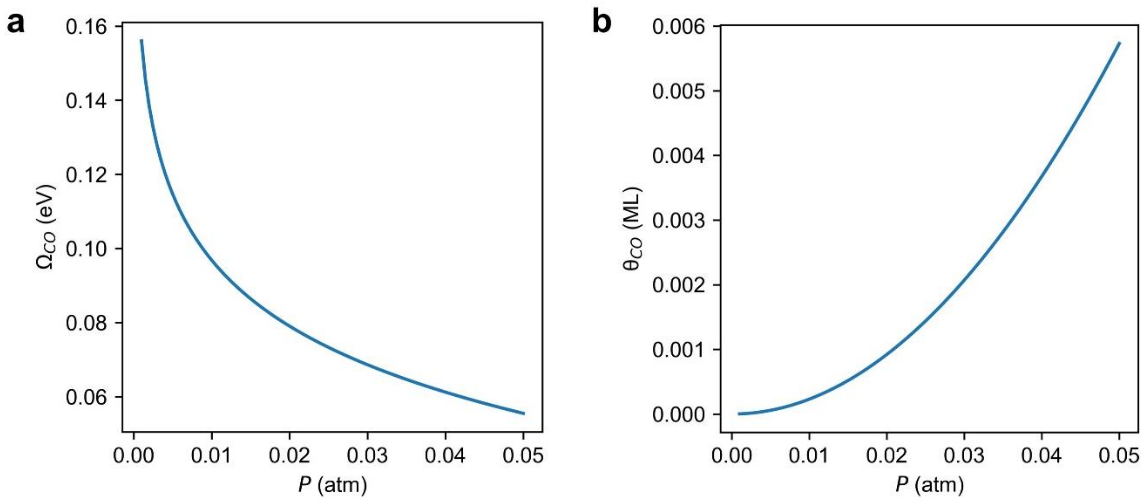

Supplementary Figure 14. (a) Adsorption free energy of CO as the function of CO pressure on Cu(100) at -1.5 V vs SHE. (b) CO coverage as the function of CO pressure on Cu(100) at -1.5 V vs SHE based on Langmuir adsorption model.

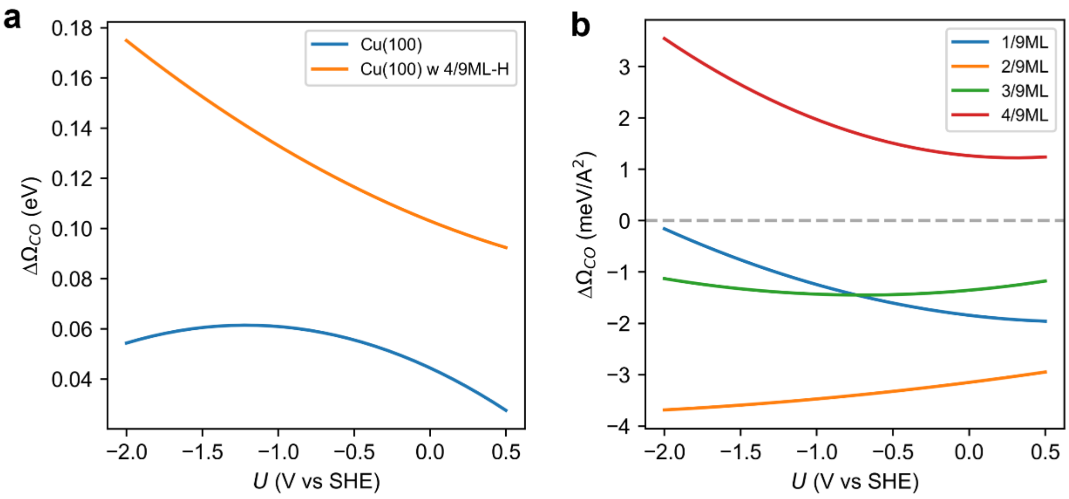

Supplementary Figure 15. Effect of H co-adsorption on CO adsorption. (a) CO adsorption energy as the function of potential on clean Cu(100) and H-covered Cu(100) surfaces. (b) Optimal CO adsorption on the H-covered Cu(711) surface.

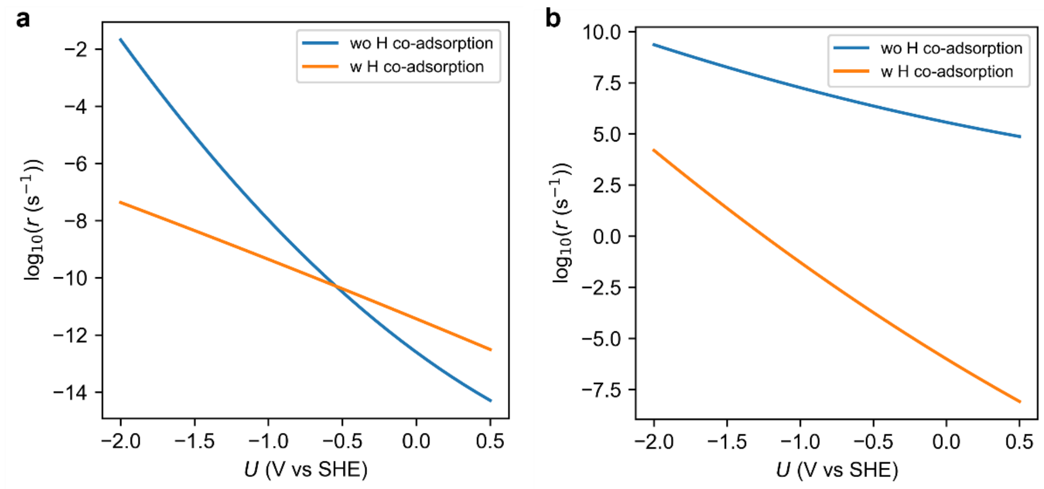

Supplementary Figure 16. Effect of H co-adsorption on reaction rate. Calculated CO_2RR rate as the function of potential on (a) clean Cu(100) and H-covered Cu(100) surfaces and (b) clean Cu(711) and H-covered Cu(711) surfaces.

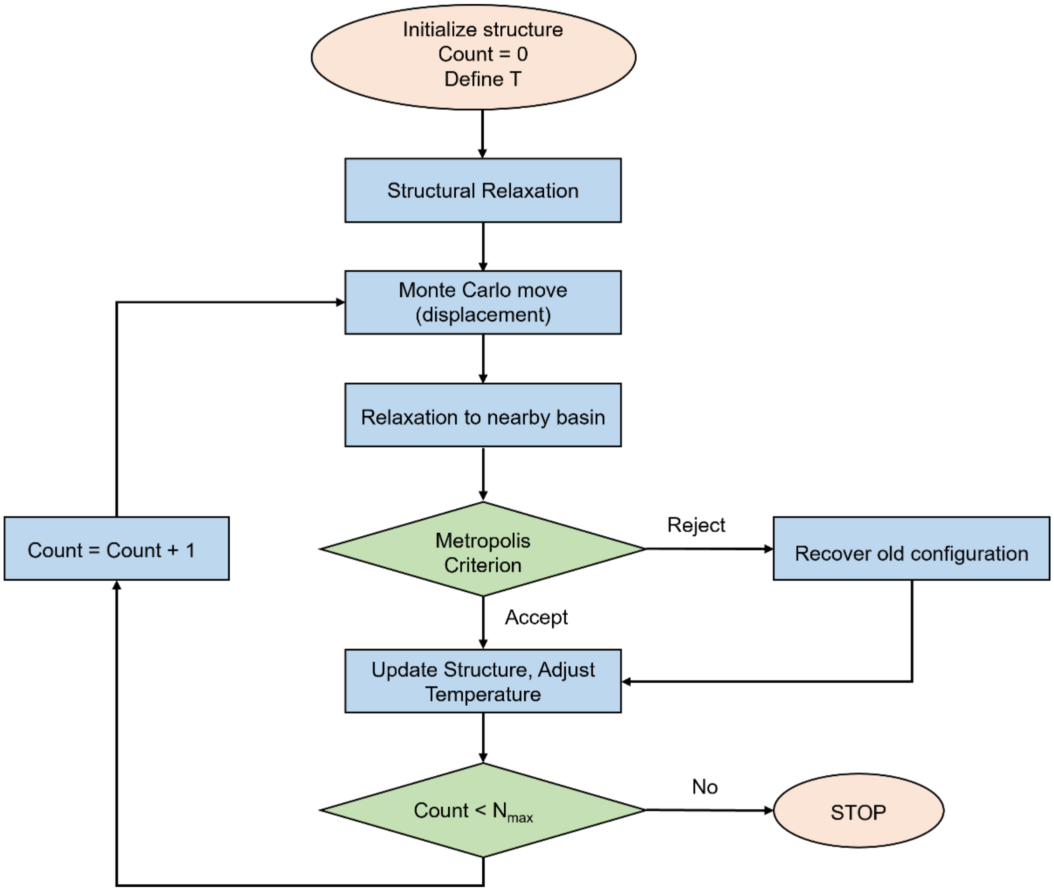

Supplementary Figure 17. Basin hopping algorithm utilized in the work.

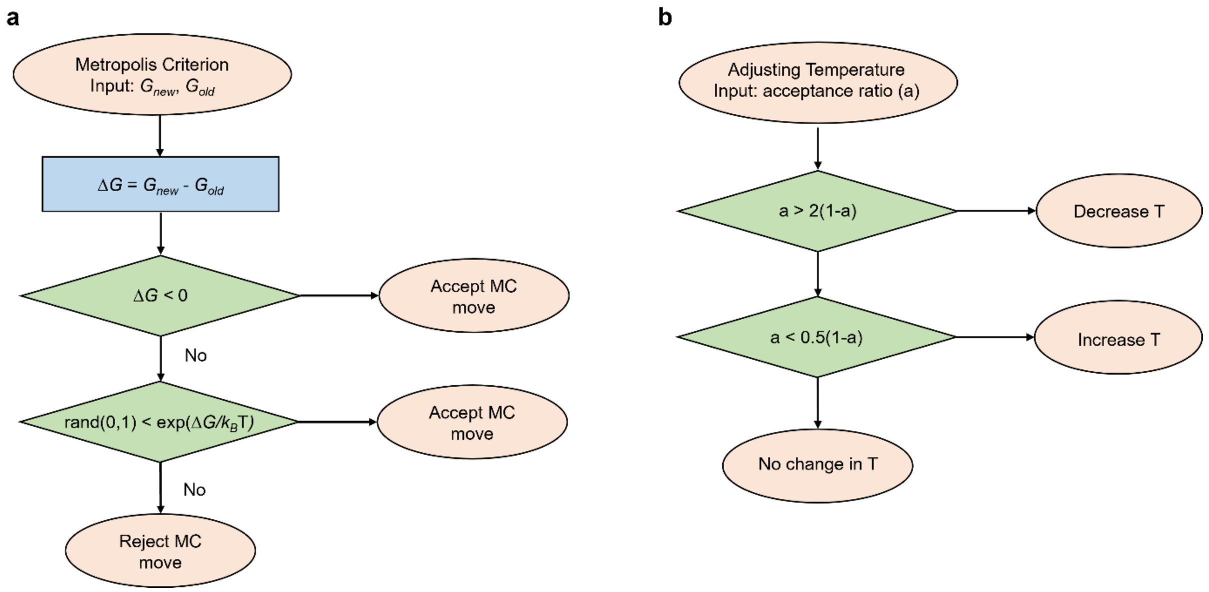

Supplementary Figure 18. Flow-chart showing the algorithm for (a) Metropolis criterion - which decides the acceptance/ rejection of MC move and (b) adjusting temperature during the BH simulation where (a) is the acceptance ratio for the BH simulation.

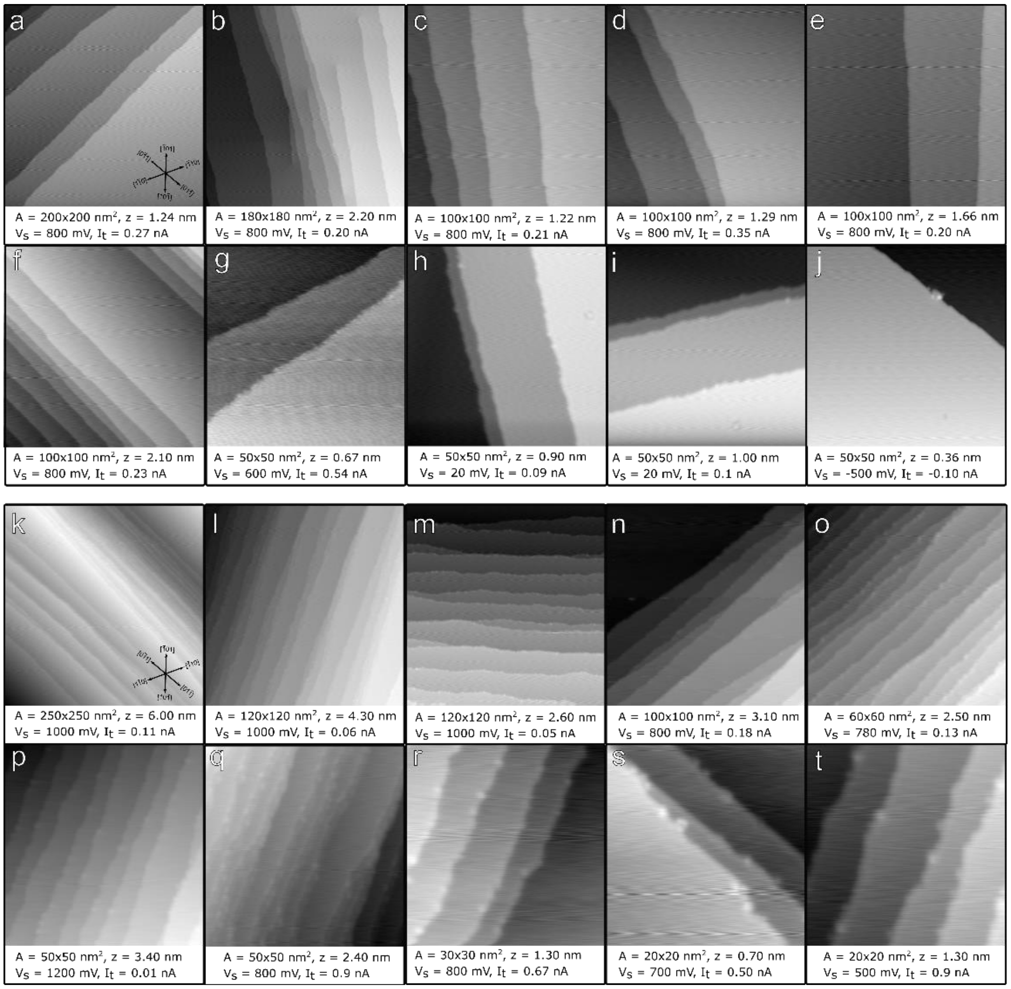

Supplementary Figure 19. Low-temperature scanning tunneling microscopy (LT-STM) images of atomically flat ultra-high vacuum (UHV)-prepared (a-j) pristine Cu(111) and (k-t) stepped Cu(111) before CO_2RR experiments. All STM images were obtained at 4K.

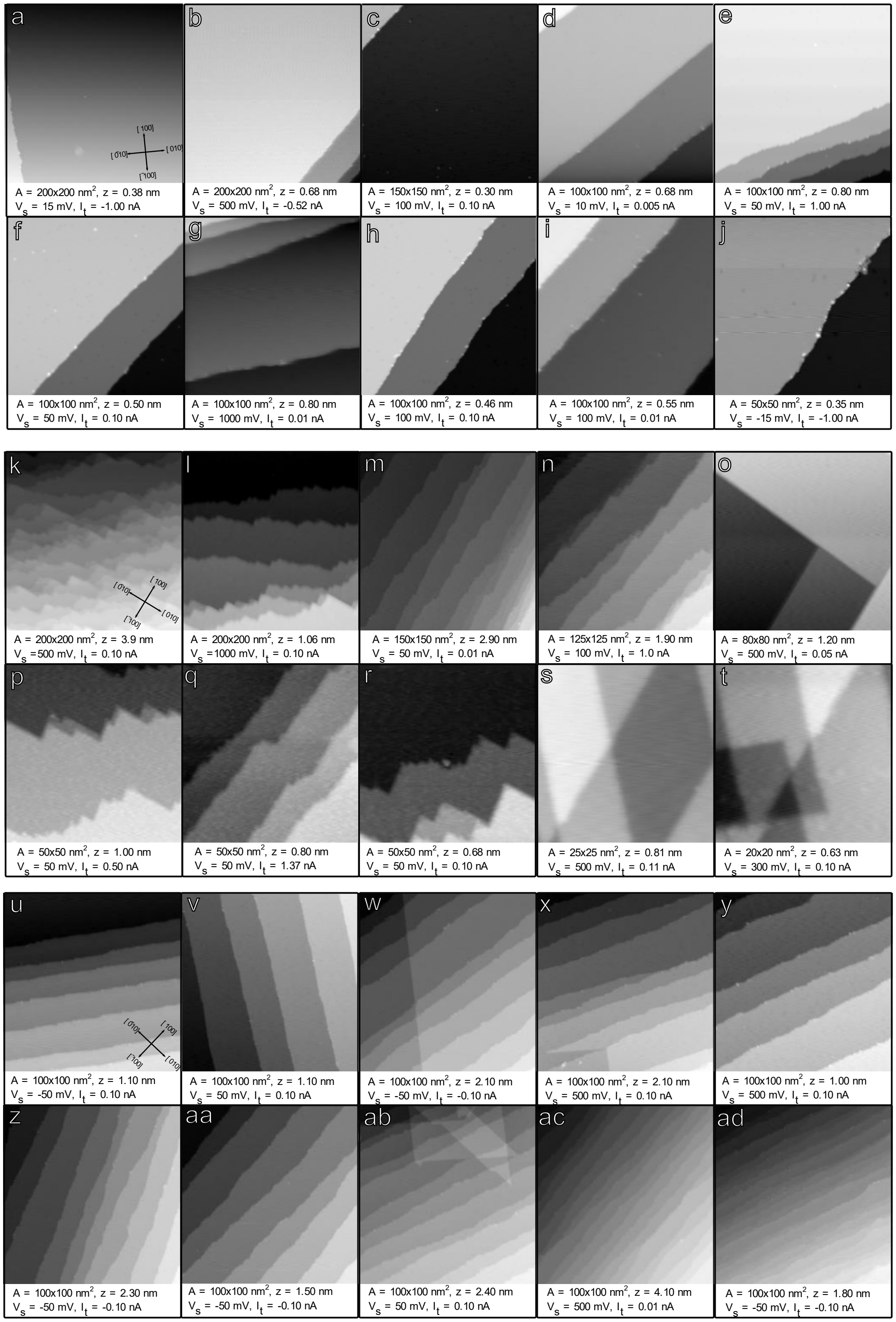

Supplementary Figure 20. Low-temperature scanning tunneling microscopy (LT-STM) images of atomically flat ultra-high vacuum (UHV)-prepared (a-j) pristine Cu(100) and (k-t) intermediate Cu(111) and (u-ad) stepped Cu(100) before CO_2RR experiments. All STM images were obtained at 4K.

Supplementary Note 1: Adsorption behavior responds to electrode potential.

In the framework of the grand canonical ensemble DFT (GCDFT), the adsorption energy of CO ($\Delta\Omega_{CO}$) on different Cu surfaces exhibits intriguing variations (Figure 1b). Notably, distinct surfaces demonstrate diverse dependencies, characterized by different curvatures and slopes, with respect to CO adsorption. For instance, Cu(843) exhibits a more linear relationship between $\Delta\Omega_{CO}$ and electrode potential (U), whereas other surfaces, such as Cu(430) and Cu(410), display more quadratic characteristics. The ($\Delta\Omega_{CO}(U)$) in the GCDFT scheme can be expressed as:

$$\Omega_{i,*}(U) = a_{i1}U^2 + b_{i1}U + c_{i1} \tag{1}$$

$$\Omega_{i,CO}(U) = a_{i2}U^2 + b_{i2}U + c_{i2} \tag{2}$$

$$\Delta\Omega_{i,CO}(U) = \Delta a_i U^2 + \Delta b_i U + \Delta c_i \tag{3}$$

$$\frac{\partial \Delta\Omega_{i,CO}(U)}{\partial U} = 2\Delta a_i U + \Delta b_i = f_1(\Delta C_H)U + f_2(\Delta C_H, \Delta pzc) \tag{4}$$

Where i means the specific surface; U represents electrode potential; a, b, c are parameters to determine the quadratic relationship of grand canonical energy (Ω) and electrode potential (U); C_H and pzc represent the Helmholtz capacitance and potential of zero charge, respectively.

It's clear that the potential-dependent adsorption energy of CO ($\Delta\Omega_{i,CO}(U)$) exhibits the quadratic relationship with U, whose characteristic was determined by the change of C_H (ΔC_H) and the change of pzc (Δpzc) upon CO adsorption, which represents the sensitivity of the adsorption energy to variations in the electrode potential. More specifically, the pzc represents the potential at which the surface is charge neutral, and its change upon CO adsorption reflects alterations in the surface charge distribution. The C_H, on the other hand, describes the response of the electrode-electrolyte interface to changes in the electrode potential. The curvature of this quadratic relationship is solely determined by ΔC_H, while the slope of the tangent line at given potential ($\frac{\partial \Delta\Omega_{i,CO}(U)}{\partial U}$) on the curve is determined by ΔC_H, Δpzc, and specific U at this point. Therefore, the distinct characteristics observed for different surfaces in Figure 1b can be attributed to the varying degrees of change in the pzc and C_H upon CO adsorption.

Supplementary Note 2: Electric field effect on the CO adsorption.

Chan's work clearly showed that the linearized Poisson−Boltzmann(LPB) implicit solvent model cannot efficiently capture the interfacial electric field effect due to the close placement of the continuum charge with respect to the surface, which will underestimate the electric field effect on the energetics[1]. We here specifically explore the field effect on CO adsorption, which is essential for us to evaluate the relative stability of various surfaces.

The interaction energy between the adsorbate and the interfacial field can be described by

$$\varepsilon = \frac{\varphi_M - \varphi_{M,PZC}}{d}$$

$$\Delta\Delta G = \mu\varepsilon - \frac{1}{2}\alpha\varepsilon^2$$

$$\Delta\Delta G = \mu\left(\frac{\varphi_M - \varphi_{M,PZC}}{d}\right) - \frac{1}{2}\alpha\varepsilon\left(\frac{\varphi_M - \varphi_{M,PZC}}{d}\right)^2$$

Where ε is the electric field, φ_M is the electrode potential and $\varphi_{M,PZC}$ represents the potential of zero charge, d is the distance between the centers of the positive charges in the Helmholtz plane and the negative charges in the surface layer in electroreduction conditions. μ and α are the dipole moment and polarizability of the adsorbate, respectively. $\Delta\Delta G$ is the change of adsorption free energy induced by the electric field.

To evaluate the effect of the chosen parameters on the stability analysis, here we calculated the dipole moment and polarizability of CO in a kinked site on the Cu(1021) surface (μ_k = -0.036 eÅ and α_k = 0.988 eÅ^2 V^{-1}, where k represents the defective kink site), which has the lowest coordination number among the surfaces we studied, and CO on planar Cu(100) surface (μ_p = 0.044 eÅ and α_p = 0.302 eÅ^2 V^{-1}, p represents planar surfaces). We expect that the states of adsorbed CO across different facets, whether on terrace sites or stepped/kinked sites, lie between these two conditions. The choice of d values is adopted from the work by Abild-Pedersen et al[2].

First, at 0.05atm, if the dipole moment (μ_k) and polarizability (α_k) from kinked CO are used, greater stabilization effects are observed under the potential of -1.5 V vs SHE on stepped/kinked surfaces (Supplementary Figure 21, 22 and 29). Since no CO can be adsorbed on the planar surfaces in equilibrium at this CO pressure (indicated in Figure 1c) , no stabilization effect is seen at this potential. We found that all the facets are more stable than Cu(111) and Cu(100) surfaces. If we use the dipole moment (μ_p) and

polarizability (α_p) from CO on close-packed planar surfaces, the trend remains consistent. We still observe a significant stabilization effect on stepped/kinked surfaces, although the stability of n(110) surfaces like Cu(430) and Cu(410) cannot compete with close-packed Cu(111) if the distance (d) is increased to 1.6 Å.

When we increase the partial pressure to 1 atm, a substantial stabilization effect is observed on stepped/kinked surfaces. Whether we use μ_k and α_k or μ_p and α_p, the stability of defective surfaces surpasses that of close-packed Cu(111) and Cu(100) surfaces, even if the distance (d) is increased to 1.6 Å (Supplementary Figure 23, 24 and 30). A similar phenomenon is seen when the CO partial pressure is increased to 60 atm (Supplementary Figure 25, 26 and 31). The strong binding of CO can reverse the stability, stabilizing defective surfaces that are not stable without CO adsorption at the potential of zero charge.

Although the absolute values will change with key parameters such as dipole moment, polarizability of *CO, and the distance between the positive and negative charge centers (d), the overall trend remains consistent. Strong CO adsorption on stepped/kinked surfaces stabilizes these surfaces, surpassing the stability of close-packed Cu(111) and Cu(100) surfaces. Therefore, CO adsorption acts as the driving force for the in-situ restructuring of planar surfaces to defective stepped and kinked surfaces under reactive conditions.

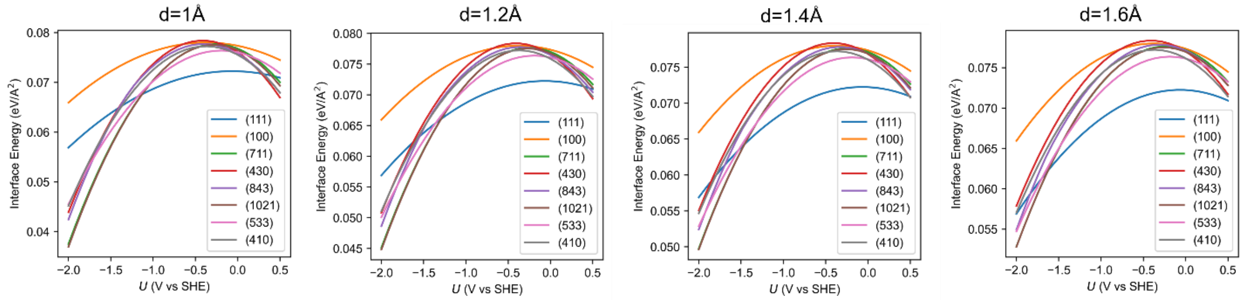

Supplementary Figure 21. Potential-dependent surface energy for the various Cu surfaces considered at 0.05 atm as the function of the distance between the positive and negative charge centers in the Helmholtz layer (d) using dipole moment and polarizability for CO adsorbed on a Cu(100) surface: μ_p = 0.044 eÅ and α_p = 0.302 eÅ² V⁻¹.

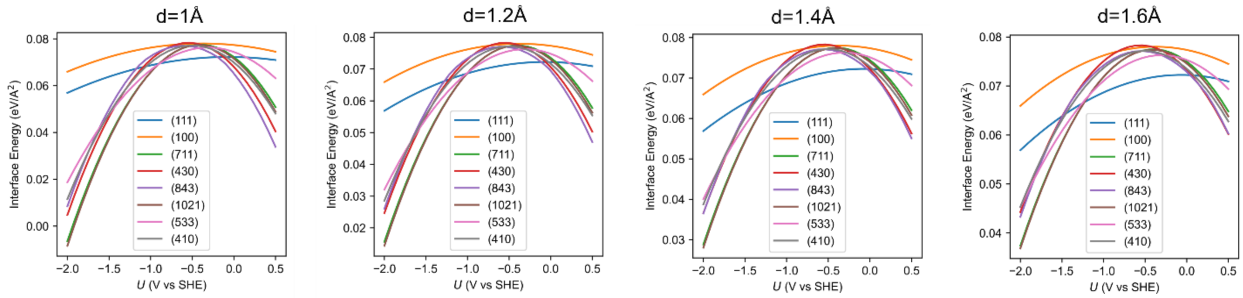

Supplementary Figure 22. Potential-dependent surface energy for the various Cu surfaces considered at 0.05 atm as the function of the distance between the positive and negative charge centers in the Helmholtz layer (*d*) using dipole moment and polarizability for CO adsorbed on a Cu(100) surface: μ_k = -0.036 eÅ and α_k = 0.988 eÅ² V⁻¹.

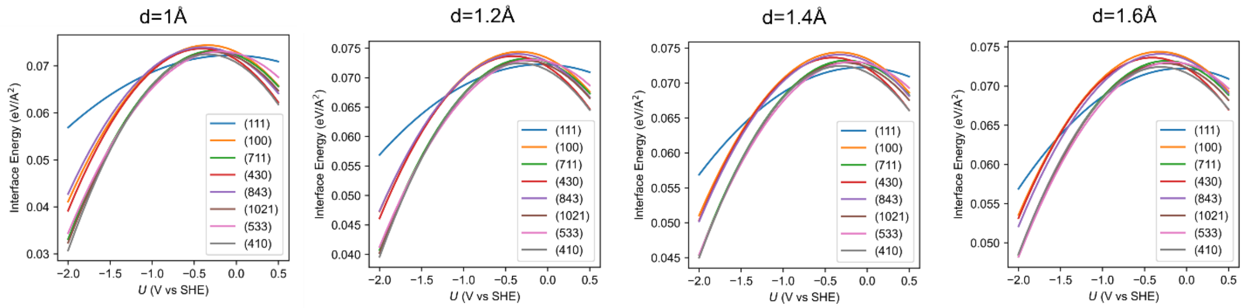

Supplementary Figure 23. Potential-dependent surface energy for the various Cu surfaces considered at 1 atm as the function of the distance between the positive and negative charge centers in the Helmholtz layer (*d*) using dipole moment and polarizability for CO adsorbed on a Cu(100) surface: μ_p = 0.044 eÅ and α_p = 0.302 eÅ² V⁻¹.

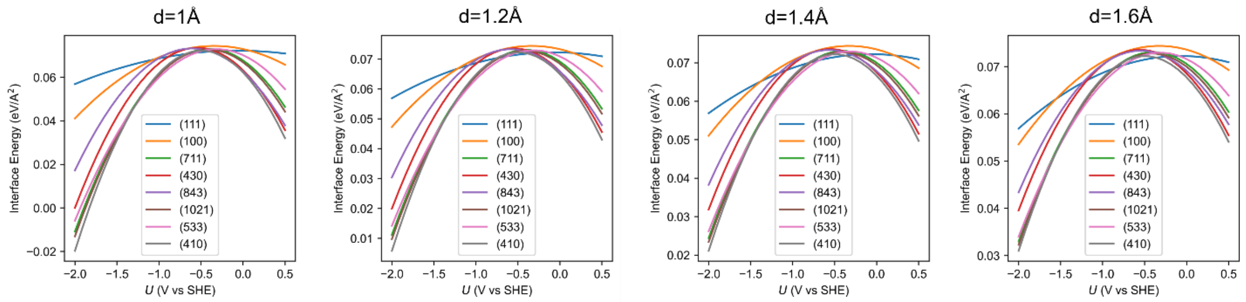

Supplementary Figure 24. Potential-dependent surface energy for the various Cu surfaces considered at 1 atm as the function of the distance between the positive and negative charge centers in the Helmholtz layer (*d*) using dipole moment and polarizability for CO adsorbed on a Cu(100) surface: μ_k = -0.036 eÅ and α_k = 0.988 eÅ² V⁻¹.

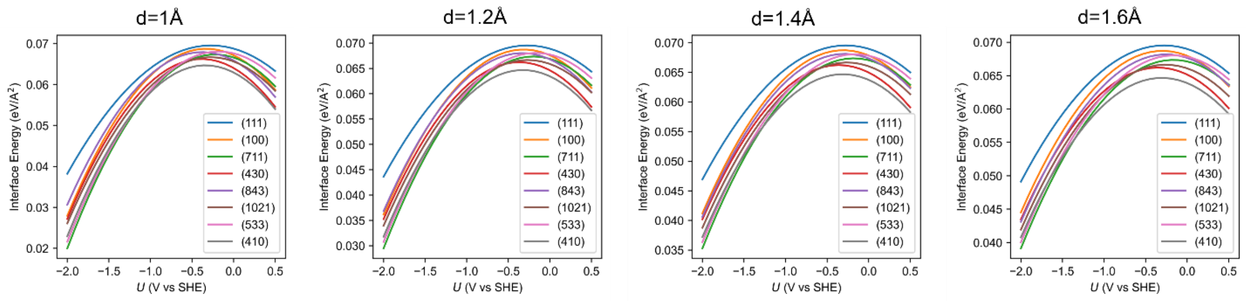

Supplementary Figure 25. Potential-dependent surface energy for the various Cu surfaces considered at 60 atm as the function of the distance between the positive and negative charge centers in the Helmholtz layer (*d*) using dipole moment and polarizability for CO adsorbed on a Cu(100) surface: $\mu_p = 0.044$ eÅ and $\alpha_p = 0.302$ eÅ² V⁻¹.

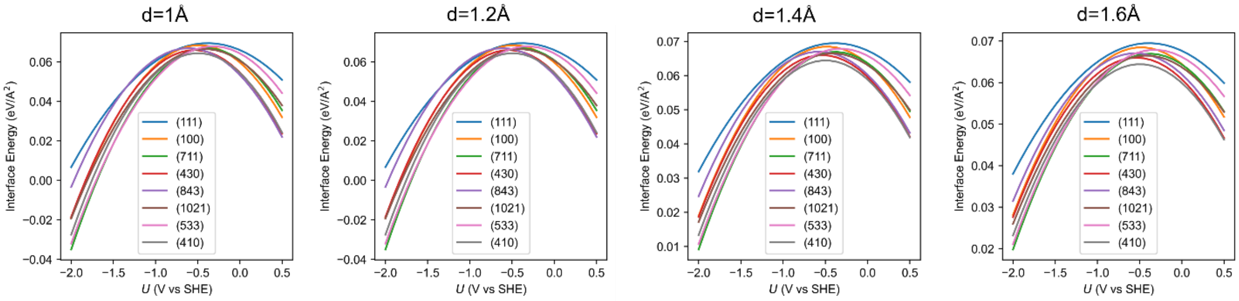

Supplementary Figure 26. Potential-dependent surface energy for the various Cu surfaces considered at 60 atm as the function of the distance between the positive and negative charge centers in the Helmholtz layer (*d*) using dipole moment and polarizability for CO adsorbed on a Cu(100) surface: $\mu_k = -0.036$ eÅ and $\alpha_k = 0.988$ eÅ² V⁻¹.

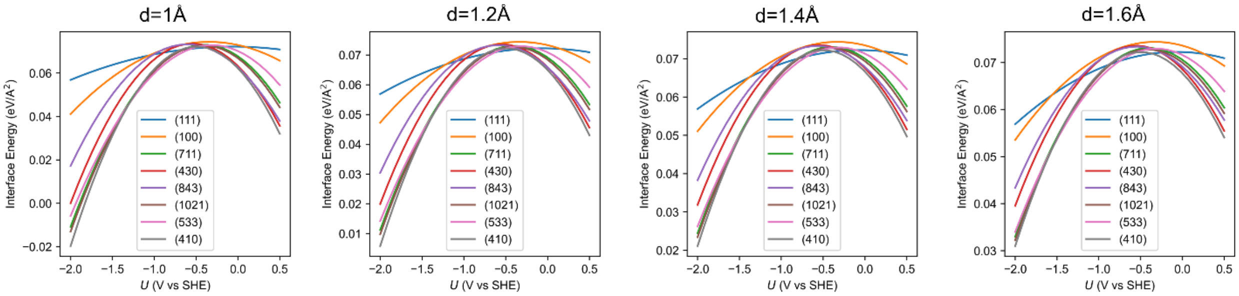

Supplementary Figure 27. Potential-dependent surface energy for the various Cu surfaces considered at 1 atm as the function of the distance between the positive and negative charge centers in the Helmholtz layer (*d*) value using dipole moment and polarizability for CO adsorbed on Cu(100) surface ($\mu_p = 0.044$ eÅ and $\alpha_p = 0.302$ eÅ² V⁻¹) for planar Cu(111) and Cu(100) surfaces and that for CO adsorbed on Cu(1021) surface ($\mu_k = -0.036$ eÅ and $\alpha_k = 0.988$ eÅ² V⁻¹) for stepped/kinked surfaces.

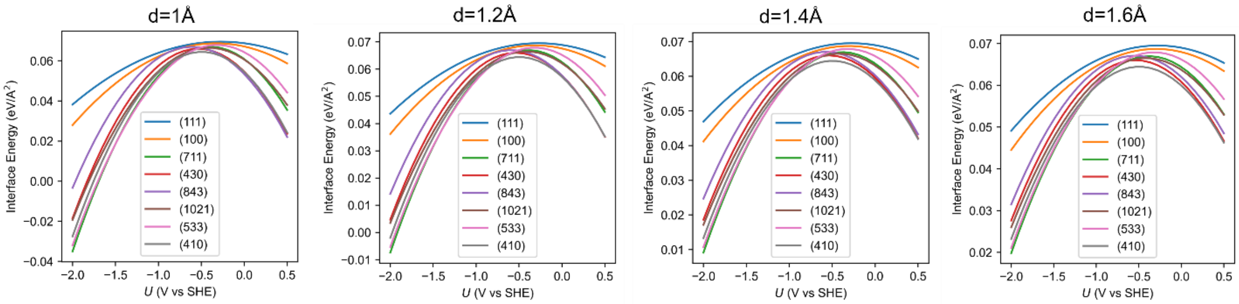

Supplementary Figure 28. Potential-dependent surface energy for the various Cu surfaces considered at 60 atm as the function of the distance between the positive and negative charge centers in the Helmholtz layer (*d*) value using dipole moment and polarizability for CO adsorbed on Cu(100) surface (μ_p = 0.044 eÅ and α_p = 0.302 eÅ² V⁻¹) for planar Cu(111) and Cu(100) surfaces and that for CO adsorbed on Cu(1021) surface (μ_k = -0.036 eÅ and α_k = 0.988 eÅ² V⁻¹) for stepped/kinked surfaces.

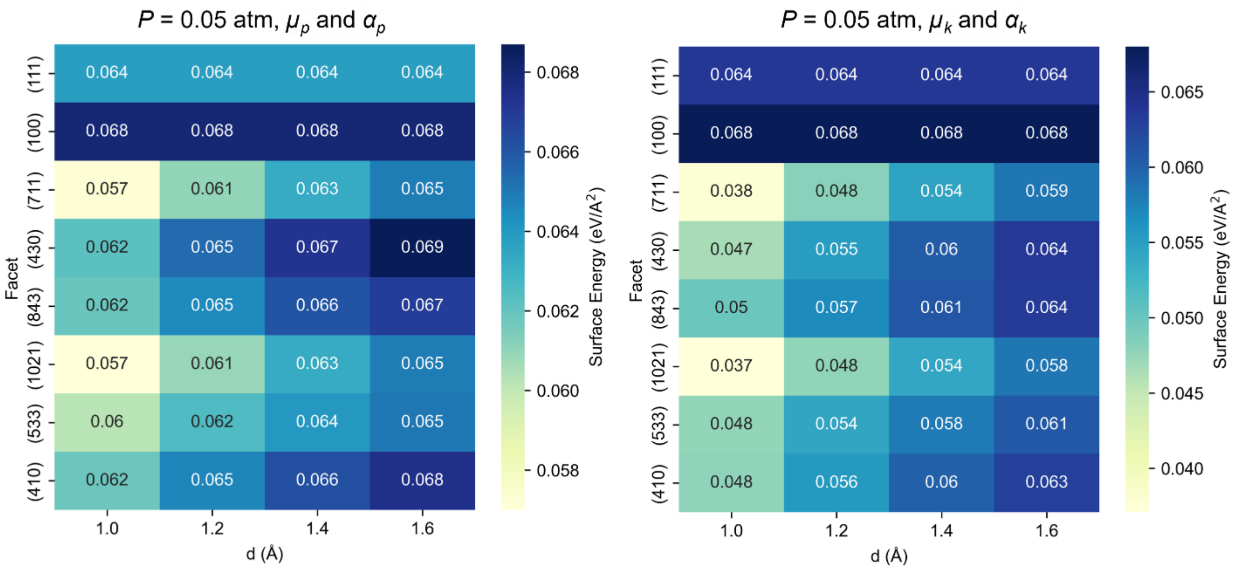

Supplementary Figure 29. Surface energy of different facets as the function of the distance between the positive and negative charge centers in the Helmholtz layer (*d*), dipole moment and polarizability for CO adsorbed on Cu(100) surface (μ_p = 0.044 eÅ and α_p = 0.302 eÅ² V⁻¹) and CO adsorbed on Cu(1021) surface ((μ_k = -0.036 eÅ and α_k = 0.988 eÅ² V⁻¹) under -1.5 V vs SHE when P_{CO} is set as 0.05 atm.

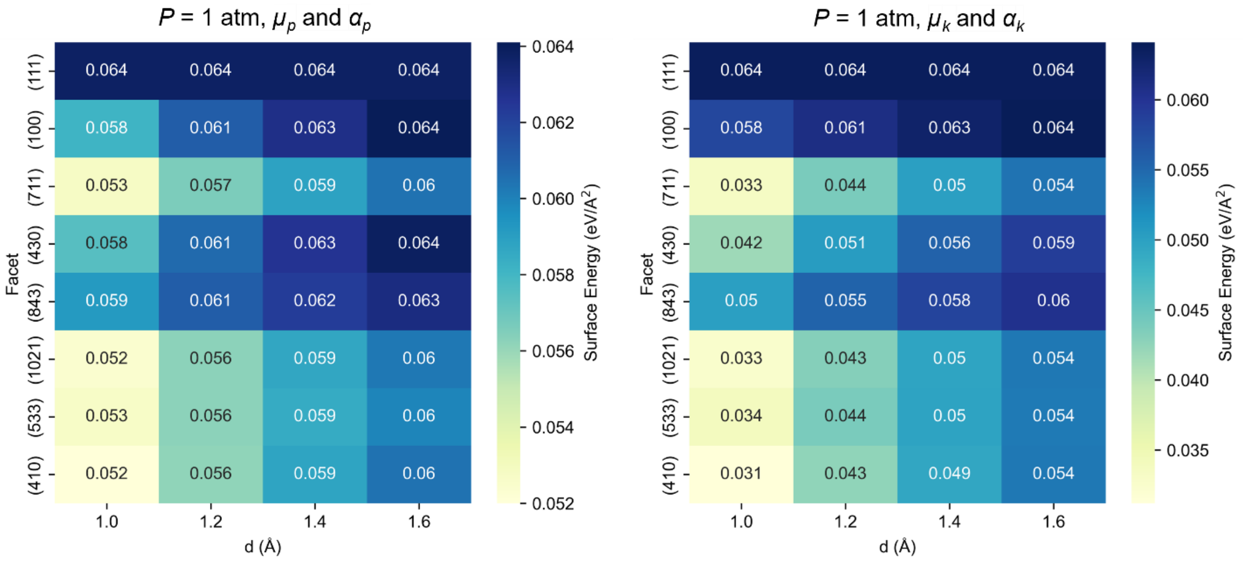

Supplementary Figure 30. Surface energy of different facets as the function of the distance between the positive and negative charge centers in the Helmholtz layer (*d*), dipole moment and polarizability for CO adsorbed on Cu(100) surface (μ_p = 0.044 eÅ and α_p = 0.302 eÅ² V^{-1}) and CO adsorbed on Cu(1021) surface ((μ_k = -0.036 eÅ and α_k = 0.988 eÅ² V^{-1}) under -1.5 V vs SHE when P_{CO} is set as 1 atm.

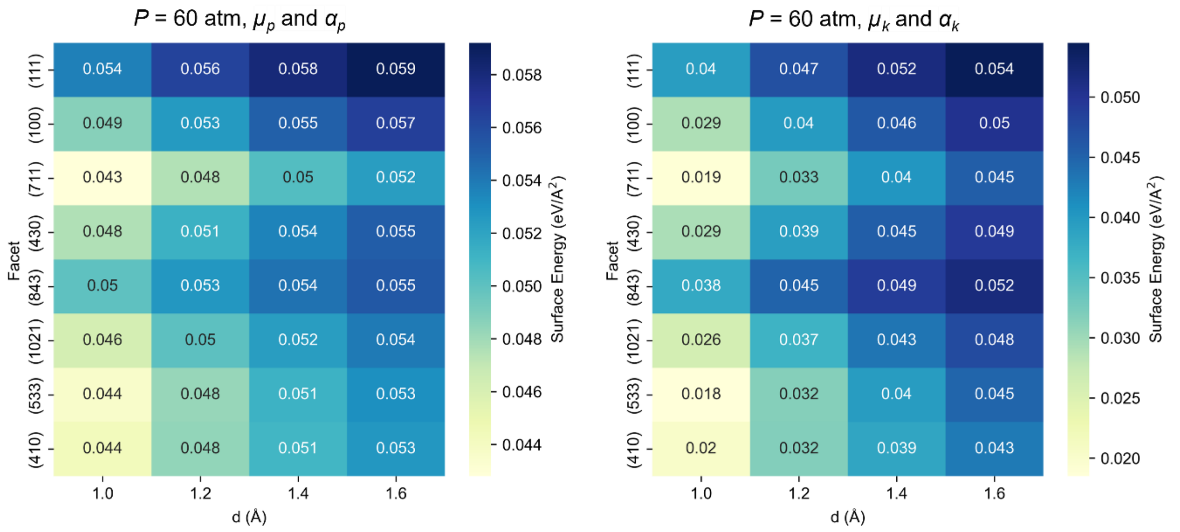

Supplementary Figure 31. Surface energy of different facets as the function of the distance between the positive and negative charge centers in the Helmholtz layer (*d*), dipole moment and polarizability for CO adsorbed on Cu(100) surface (μ_p = 0.044 eÅ and α_p = 0.302 eÅ² V^{-1}) and CO adsorbed on Cu(1021) surface (μ_k = -0.036 eÅ and α_k = 0.988 eÅ² V^{-1}) under -1.5 V vs SHE when P_{CO} is set as 60 atm.

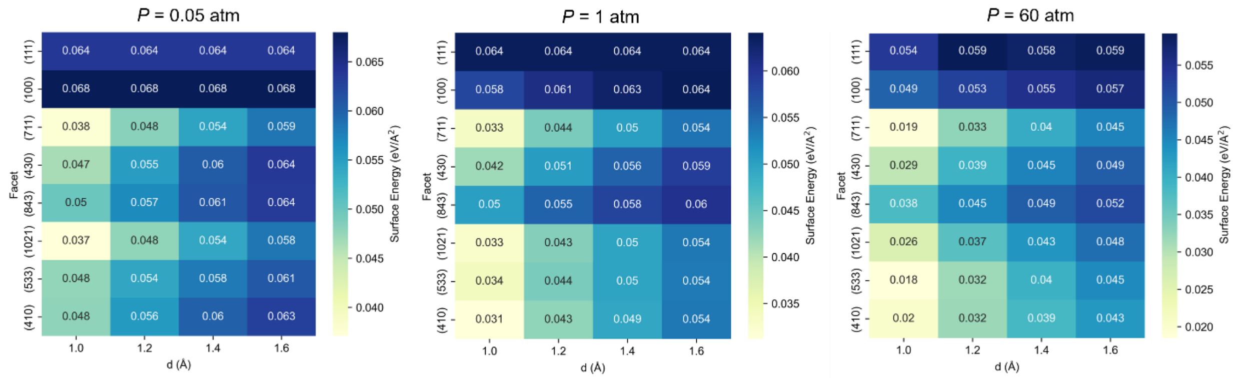

Supplementary Figure 32. Surface energy of different facets as the function of the distance between the positive and negative charge centers in the Helmholtz layer (*d*) *and* CO partial pressure under -1.5 V vs SHE using dipole moment and polarizability for CO adsorbed on Cu(100) surface (μ_p = 0.044 eÅ and α_p = 0.302 eÅ² V^{-1}) for planar Cu(111) and Cu(100) surfaces and that for CO adsorbed on Cu(1021) surface (μ_k = -0.036 eÅ and α_k = 0.988 eÅ² V^{-1}) for stepped/kinked surfaces.

Supplementary Note 3: The effect of subsurface oxygen on CO_2RR.

The existence of subsurface O is still the subject of intense debate, due in part to the experimental challenges of detecting small amounts of oxygen in Cu or highly disordered Cu oxides/hydroxides under operando reaction conditions. Some early studies claimed that oxygen species in the surface and subsurface of the Cu catalyst are key for achieving high activity and hydrocarbon/alcohol selectivity. Goddard's group made significant theoretical contributions to exploring the role of subsurface oxygen in CO_2 adsorption and C-C coupling. They concluded that subsurface O could promote CO_2 activation and CO dimerization[3]. A very recent paper from López's group claimed that it would take some time to completely consume the subsurface oxygen on oxide-derived Cu[4]. However, other theory and experimental studies have challenged this hypothesis, even though the detection of very small amounts of subsurface oxygen would be beyond the sensitivity limits of the experimental methods employed in some of the former studies. On the theory side, Bell and Head-Gordon et al concluded from DFT calculations that subsurface O species are unstable and do not provide an increased activity, compared to purely metallic sites[5]. Chan and Norskov et al also reported that no oxide will remain at the surface or promote the adsorption of CO_2 intermediates[6]. Nonetheless, both studies employed ideal "flat" metallic surfaces as a starting point, and we know experimentally that the most active and C-C coupling selective Cu surfaces are not ultrapure Cu surfaces, but highly defective Cu surfaces, where the stability of oxygen or surface/subsurface oxides would be very different, and where dissolved oxygen is present in small amounts in the bulk and might serve as oxygen reservoir. The same consideration also applies to the commonly employed oxide-derived Cu surfaces or bulk-like Cu_2O electrocatalysts, which are more likely to retain oxygen during longer reaction times. The presence of O on Cu surfaces in the presence of hydroxides is also reflected in the cathodic corrosion that has been experimentally observed with increasing reaction times, with some $CuOH_x$ species becoming gradually dissolved in the alkaline electrolyte.

On the other hand, the favorable role of oxygen-containing Cu electrocatalysts in CO_2RR for the production of oxygenates (e.g. ethanol) has been evidenced in multiple quasi-*in situ* and *operando* studies from the Roldan group[7-10], including those where oxidative potential pulses were used to re-generate the surface oxides/hydroxides. Some of the prior discussion in the literature related to whether there is a positive catalytic effect in the presence of cationic Cu species might be reconciled based on the work from Timoshenko et al.[8], which, under potentiodynamic reaction conditions, unveiled C1-products being generated on bulk-like Cu_2O surfaces, while ethanol being obtained on surfaces containing highly disordered Cu/Cu(I)/Cu(II) species.

Thus, based on the collective information from both experimental and theoretical aspects, we hypothesize that for certain Cu-based catalysts, such as oxide-derived Cu (OD-Cu) and Cu_2O pre-catalysts, or very

rough Cu surfaces during short reaction times, subsurface oxygen could contribute to CO_2 activation and C-C coupling at the beginning of CO_2RR. However, the lifetime of subsurface oxygen during CO_2RR under a constant negative applied potential should be short, and ultimately, the surface structures formed during the reaction will likely play a more crucial role in determining CO_2RR activity.

Supplementary Note 4: The effect of dielectric constant on CO adsorption behavior.

The dielectric constant of water near the surface is much lower than that in bulk and approximating the dielectric constant as 78.4 may not always accurately describe the adsorption of intermediates. We conducted benchmarks on CO adsorption, as well as the important physical properties including double-layer capacitance and the potential of zero charge as functions of the dielectric constant.

For the Cu(100) surface, we observed that decreasing the dielectric constant from 78.4 to 1 (vacuum) makes the adsorption energy more positive due to the reduced solvent stabilization effect. However, the adsorption energy difference of CO between a dielectric constant of 78.4 and 1 is only 0.019 eV, indicating that the solvent effect on CO adsorption is negligible (Supplementary Figure 33). We also performed RPA calculations on CO adsorption with and without the linearized Poisson–Boltzmann equation to describe the solvation effect. Compared to the vacuum condition, the energy difference was similarly small, at 0.030 eV. This trend was consistent on the Cu(711) surface, where the difference was just 0.017 eV (Supplementary Figure 34). These findings suggest that the effect of dielectric constant on CO adsorption is minimal, regardless of the surface chosen, and does not influence our results.

Furthermore, when the dielectric constant is set to 78.4, the double-layer capacitance and the potential of zero charge closely match experimental values. Conversely, a lower dielectric constant sharply reduces the double-layer capacitance and shifts the potential of zero charge to more positive values, leading to unphysical changes in potential-dependent energetics (Supplementary Figure 33). Therefore, considering the very small solvent effect on CO adsorption, maintaining realistic electrochemical interface properties by using the default dielectric constant of 78.4 is more important for our grand canonical DFT calculations. Addressing how to accurately describe the near-surface region using the implicit solvent model remains an interesting topic for future research.

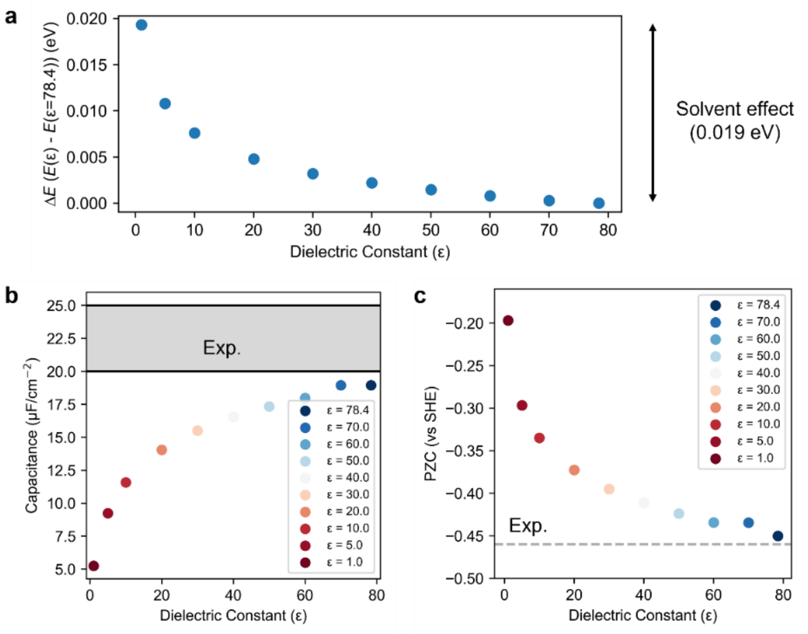

Supplementary Figure 33. The effect of dielectric constant on Cu(100) surface. (a) Relative adsorption energy of CO as the function of dielectric constant. (b) Calculated double-layer capacitance and (c) potential of zero charge as the function of dielectric constant. Experimental values are represented by (b) shaded region and (c)dotted line.

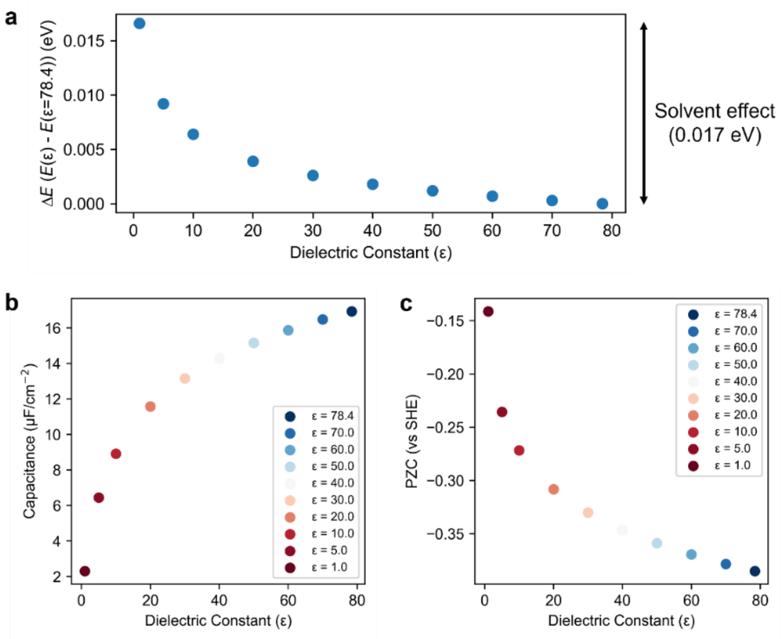

Supplementary Figure 34. The effect of dielectric constant on Cu(711) surface. (a) Relative adsorption energy of CO as the function of dielectric constant. (b) Calculated double-layer capacitance and (c) potential of zero charge as the function of dielectric constant.

Supplementary Note 5: The rate-determining step of CO_2 conversion to CO on Cu.

In terms of CO_2 conversion to CO, both $*CO_2$ and *COOH adsorption can serve as descriptors, as discussed in Chan's work[15]. They proposed that if the adsorption energy of $*CO_2$ is stronger than that of *COOH, then ΔG(*COOH) would be decisive. Conversely, if $\Delta G(*CO_2)$ is weaker than ΔG(*COOH), then CO_2 adsorption would be the rate-determining step.

We compared the adsorption energy of $*CO_2$ and *COOH (bare surface and gas molecules as the reference) across different Cu facets. We found that the formation energy of *COOH exhibits scaling relationships with respect to general coordination number (GCN) across various facets as well, the same as that of $*CO_2$. Low-coordination sites effectively promote the reduction of $CO_2(g)$ to *COOH, indicating that similar to $*CO_2$ adsorption, reduction to adsorbed *COOH could also serve as a good descriptor for the structural sensitivity of CO_2 conversion to CO (Supplementary Figure 35. a).

However, at relatively negative potentials, the formation energy of *COOH on various facets is negative, indicating that *COOH formation is favorable across all facets, including the inert planar Cu(111) and Cu(100) surfaces. When comparing the formation energies of $*CO_2$ and *COOH, we observed that $*CO_2$ adsorption generally is less energetically favored compared to *COOH formation. This implies that in most cases, especially within the potential range of interest (-1.0 V to -1.5 V vs SHE), $*CO_2$ adsorption would be the rate-determining step across all facets in the CO_2 conversion to CO process (Supplementary Figure 35. b).

While the existing linear relationship between $*CO_2$ and *COOH suggests that *COOH adsorption could provide a qualitative trend, choosing $*CO_2$ adsorption as the descriptor seems more accurate and physically meaningful.

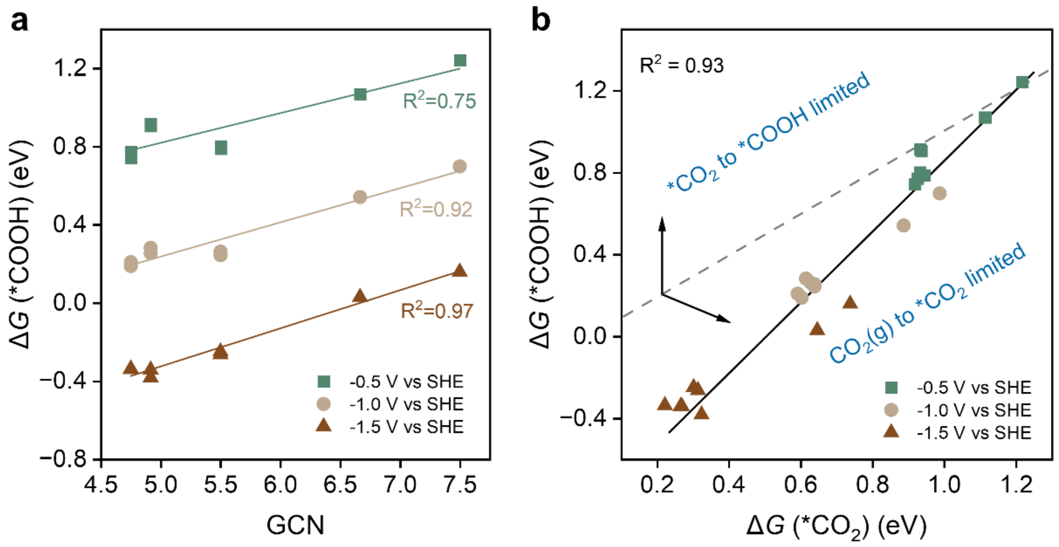

Supplementary Figure 35. Rate-determining step of CO_2RR to CO across Cu facets (a) Adsorption free energy of *COOH as the function of general coordination number(GCN) and electrode potential. (b) Comparison of adsorption free energy of *CO_2 and *COOH across different Cu facets under different electrode potentials. The dashed line indicated the identical adsorption energy of *COOH and *CO_2. Arrows indicate the rate-determining steps.

Supplementary Note 6: The choice of experimental partial pressure of CO in kinetics modeling.

A full micro-kinetics modelling is a good way to determine *CO coverages. However, one should underline that microkinetic modeling needs highly accurate reaction barriers for each elementary step. However, current technologies to determine the proton-coupled electron transfer barriers are still limited, especially considering its potential-dependent nature and the choice of explicit solvent models. It should be noted that a small fluctuation of reaction barriers would lead to a huge change in reaction rate, considering the intrinsic error of adsorption energy in DFT, 0.15 eV, the shift in reaction barrier of this magnitude gives a 300 change in the TOF at room temperature, which is emphasized by Chan et al[16]. Furthermore, adsorbed *CO will be in equilibrium with dissolved CO in the solvent, which needs an additional mass transport model to be described. Unfortunately, given the complex solvent environment of the double layer, such mass transport effect is very hard to accurately model. Thus, DFT-based kinetics models currently cannot output the quantitative results of surface coverage of intermediates, reaction rate, and selectivity, especially when compared to the experiments where mass transport and surface morphology are well controlled. Given the above reasons, we directly chose the CO pressure in our kinetics models from well-controlled Cu single-crystal experiments from Roldan et al.

Supplementary Note 7: The cation effect on the surface stability and CO_2RR activity.

Some hypotheses have been proposed that cations can tune the proton concentration near the electrode or regulate the local CO_2 concentration. However, cation effects are present at sufficiently low electrode potentials where the mass transport limitations are minimized[17], which suggests the field induced by cations would be an important factor in tuning the adsorption of key intermediates in CO_2RR, as validated by both experiments and theories[18]. Early works employed field-dipole theory to represent the cation effect and quantitively reproduced the experimental activity and selectivity[19, 20]. The linearized Poisson−Boltzmann solvent (LPB) model employed in this work places the continuum charge extremely close to the surface, causing the electric field along the z-axis to decay quickly. Consequently, the LPB model would underestimate the electric field effect[1]. To evaluate the cation effect in the activity and surface stability, we employed field-dipole theory, since directly modeling it, especially in combination with LPB, would face some technical problems, for example, unphysical invasion of continuum charges into the space of the explicit solvent.

First, we calculated the dipole moment and polarizability of *OC-CO(TS) across different Cu facets and different sites, and we found that the effect of local coordination is rather small (Supplementary Figure 36). Thus, we used the calculated dipole moment and polarizability of *OC-CO(TS) on the Cu (711) step-edge sites to represent that on steps/kinks. Two additional effects need to be mentioned. First, as discussed in Supplementary note 4, the dielectric constant near the surface would be much smaller than that in the bulk, leading to an overestimation of the solvent effect on the adsorbates. We calculated the adsorption energy of *OC-CO(TS) as the function of the dielectric constant and found that a correction of 0.17 eV is necessary when the dielectric constant is 5 (Supplementary Figure 37). Second, GGA-level functionals tend to overestimate the CO binding energy on bridge or hollow sites due to the artificial strengthening of the 2π-d back-bonding interactions. Since *OCCO and *OC-CO(TS) are both adsorbed on bridge/hollow sites, it is essential to correct the energy against RPA values. Our previous work found that RPBE functional overestimates the binding energy by 0.36 eV compared to RPA values on *OCCO adsorption[21]. We here assume the energy correction for *OCCO and *OC-CO(TS) is the same. Therefore, the overall correction on *OC-CO (ΔE) should be expressed as:

$$\Delta E = \Delta E(\text{field-dipole-corr}) + \Delta E(\epsilon\text{-corr}) + \Delta E(\text{RPA-corr})$$

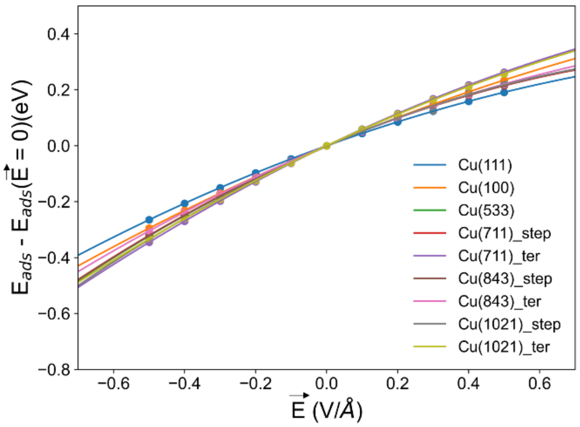

Supplementary Figure 36. Adsorption energy of *OC-CO (TS) as a function of the electric field strength on different Cu facets.

Supplementary Table 1: The calculated dipole moment (μ) and polarizability(α) of *OC-CO on different local structures.

Site	μ (eÅ)	α (eÅ² V⁻¹)
Cu(111)	0.46	0.29
Cu(100)	0.53	0.24
Cu(533)	0.54	0.43
Cu(711)_step	0.54	0.42
Cu(711)_ter	0.59	0.33
Cu(843)_step	0.54	0.42
Cu(843)_ter	0.53	0.34
Cu(1021)_step	0.55	0.45
Cu(1021)_ter	0.59	0.30

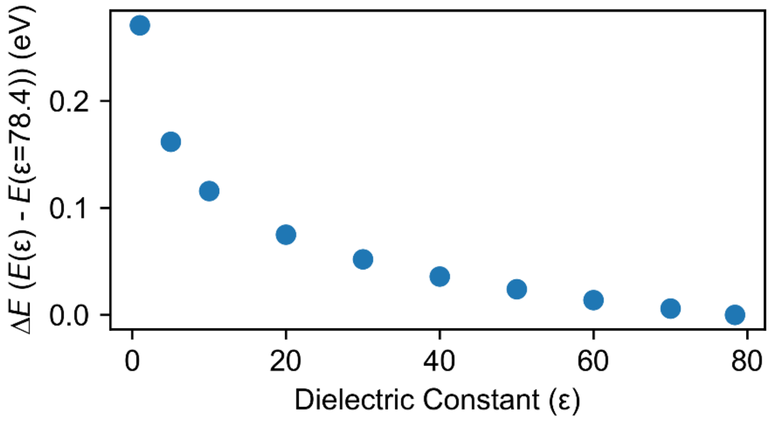

Supplementary Figure 37. Adsorption energy of *OC-CO as a function of the dielectric constant on Cu(100) surface.

After applying the field-dipole correction, stabilization effects are observed on almost all sites. Nevertheless, the trend remains unchanged: square sites show a preference for C-C coupling, while on triangle sites and stepped sites, C-C coupling barriers are significantly increased due to the unfavorable binding mode for *OCCO (Supplementary Figure 38). We further evaluated the kinetic data with the field correction. We found that after the field correction, the reaction rates increased by about an order of magnitude on all surfaces due to the decreased C-C coupling barriers (Supplementary Figure 39). However, the Cu(111) and Cu(100) surfaces still show almost no activity for CO_2RR to C2 products due to the limited CO coverage. As for the stepped or kinked surfaces, Cu(711), Cu(410), and Cu(1021) still exhibit much higher activity than the other surfaces due to the presence of square sites. Thus, applying the field correction does not change the overall trend of the activity analysis. Moreover, the similar dipole moment (μ) and polarizability(α) of *OC-CO (TS) adsorbed across the various surfaces show that variations in field strength will lead to comparable changes in the adsorption energy of *OC-CO on these surfaces. Consequently, tuning the cations (adjusting the strength of the electric field) will not alter the relative trends observed.

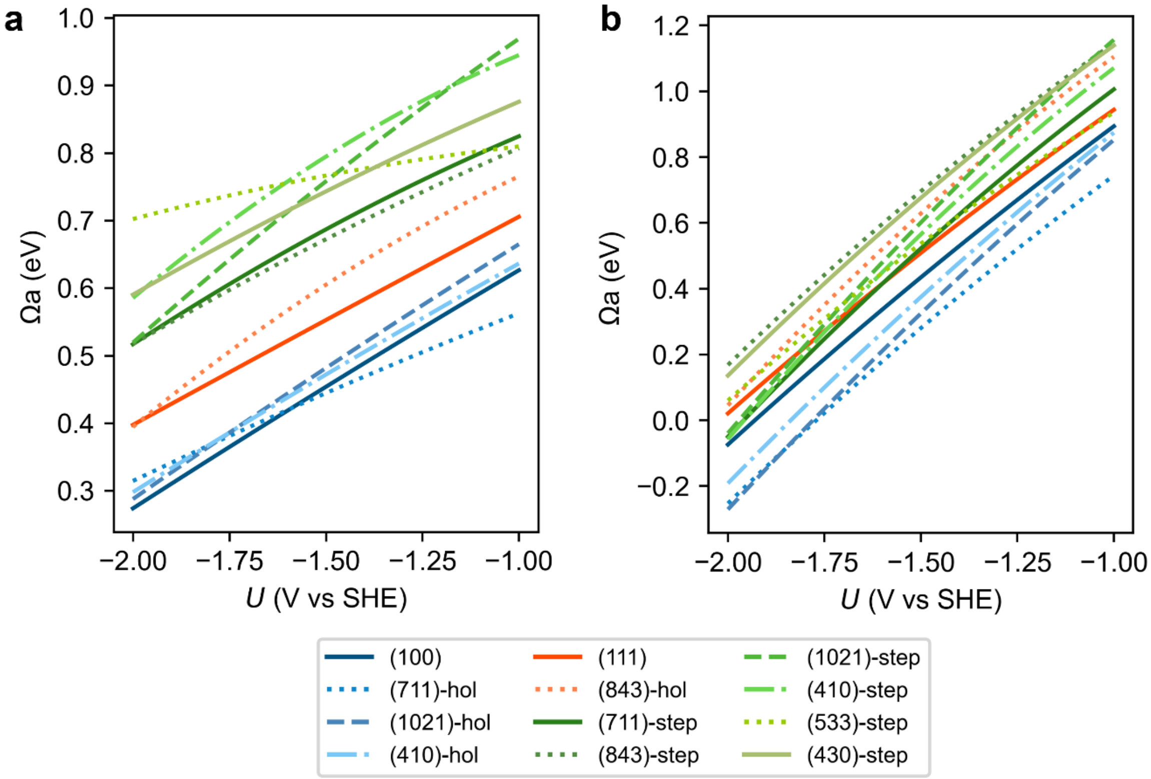

Supplementary Figure 38. CO-CO coupling barrier on different Cu surfaces as the function of potential (a)w/o field correction (b) with field correction.

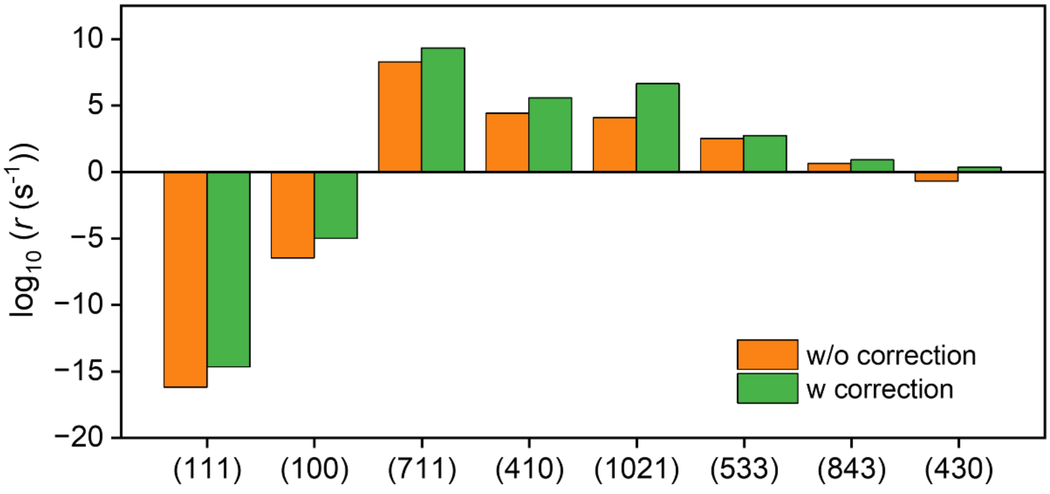

Supplementary Figure 39. Calculated reaction rate for CO_2RR to C2 products on different Cu surfaces as the function of potential with and without field correction.

Regarding *CO_2 adsorption, it is not directly associated with the final activity analysis. We want to emphasize the significance of defective sites for CO_2 activation, which precedes the rate-determining step, C-C coupling, and is crucial for achieving sufficient CO coverage. Therefore, we focus on highlighting the qualitative trend of CO_2 adsorption on various sites.

We tested the dipole moment (μ) and polarizability (α) on Cu(100) and Cu(711) and found that the values are similar (Supplementary Figure 40). This indicates that similar to the case of *OC-CO(TS), these properties are not sensitive to the local coordination of Cu. Nevertheless, to ensure greater accuracy, we used the μ and α from the Cu(100) surface to represent planar surfaces and those from the Cu(711) surface to represent defected surfaces. We compared *CO_2 adsorption with and without field correction on different sites at -1.0 V vs SHE, which is near the onset potential for CO production in CO_2RR. We found that the field can stabilize *CO_2 on all surfaces; however, the trend remains unchanged (Supplementary Figure 41). Planar Cu(111) and Cu(100) surfaces are not conducive for CO_2 activation even at relatively negative potentials, while defective sites can effectively activate CO_2 and promote CO_2 conversion. Although we did not consider the energy correction arising from the difference in dielectric constant between the interface and the bulk, the constant correction ensures that the trend remains unchanged.

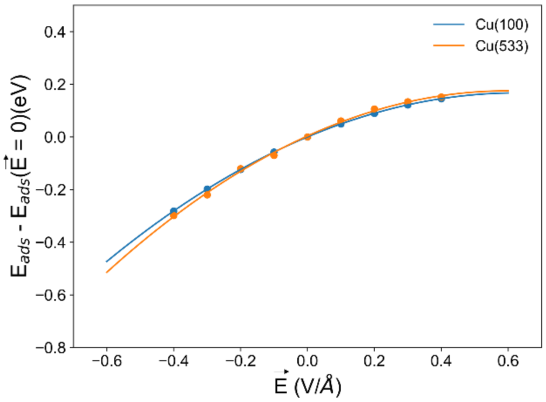

Supplementary Figure 40. Adsorption energy of *CO_2 as a function of the electric field strength on Cu(100) and Cu(711) facets.

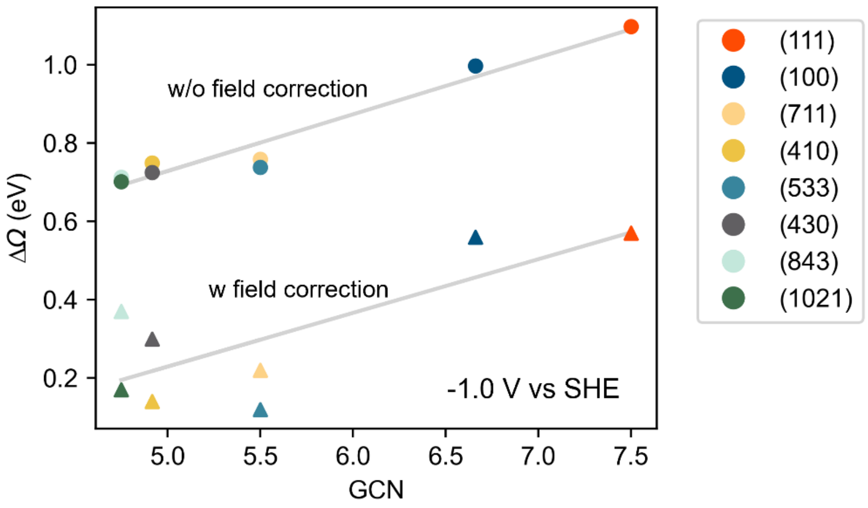

Supplementary Figure 41. Adsorption energy of $*CO_2$ as a function of the general coordination number (GCN) at -1.0 V vs SHE.

For details of the surface stability analysis with field-dipole interaction, see **Supplementary Note 2**.

In summary, the calculations demonstrated that the electric field generated by the electrolyte cations in the vicinity of the electrode is important in CO_2RR, particularly in influencing the energetics of intermediates with large dipole moments. However, we observed that the dipole moment and polarizability of key intermediates ($*CO_2$ and *OCCO) are similar across various surfaces, indicating that the field-induced changes are consistent across different surfaces. This suggests that tuning the cations (altering the field strength) will not impact the relative trends observed in our calculations. Therefore, we conclude that whether or not the cation effect is included, the main conclusions of our work remain unchanged. The thermodynamic stability trends under reaction conditions are consistent, as is the corresponding activity trend.

Moreover, the hybrid model, which includes explicit water molecules and cations, along with an implicit solvent model to represent the bulk solvent, could be a good choice for further theoretical studies. However, it currently faces technical challenges such as the invasion of the continuum charges and extensive sampling of local solvent structures. In addition, we acknowledge the potential for non-electric field (NEF) effects, such as local interactions presented by large cations. Accurately modeling these interactions requires ab initio molecular dynamics (AIMD) with fixed electrode potential and long timescales to capture the complexities of electrochemical processes and the dynamics of the solvent and electrolyte. While such a comprehensive modeling would be valuable, it is beyond the scope of this work and remains an interesting avenue for future research.

Supplementary Note 8: The competition with H_2O adsorption

In reactive conditions, H_2O may compete with CO in adsorption, thus affecting the CO coverage. We selected Cu(100), Cu(711), and Cu(1021) as examples of representative planar, step, and kink surfaces. We compared CO and H_2O adsorption as a function of the electrode potential. Our findings indicate that CO adsorption is relatively insensitive to the electrode potential, while H_2O adsorption shows a strong dependence on potential (Supplementary Figure 42). At very positive potentials, H_2O adsorption is stronger than CO adsorption. However, as the potential becomes more negative, H_2O adsorption weakens sharply, while CO adsorption remains nearly unchanged. The crossovers of adsorption energy between H_2O and CO on Cu(100), Cu(711), and Cu(1021) surfaces occur at 0 V, 0.08 V, and -0.16 V vs SHE, respectively. This indicates that in the negative potential region, H_2O adsorption cannot compete with CO adsorption. Especially within the CO_2RR potential range of -1.0 V to -1.5V vs SHE, the energy differences exceed 0.5 eV. Additionally, experimental results show that at negative potentials, the H_2O layer adopts an H-down configuration, which prevents H_2O molecules from adsorbing onto the surface[25]. Therefore, we can exclude the competition from H_2O adsorption within the potential range we are interested in.

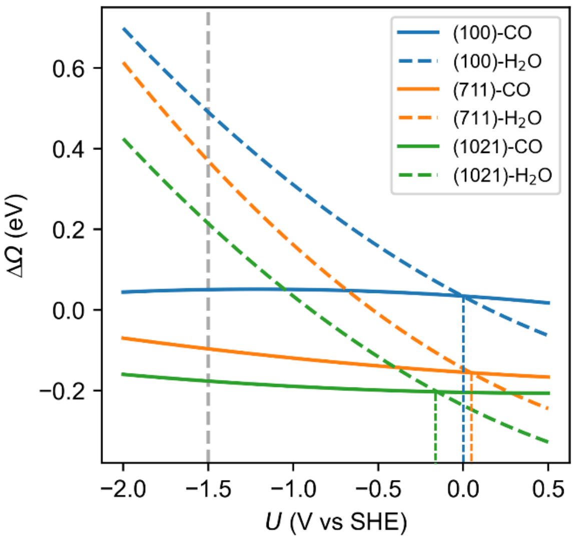

Supplementary Figure 42. The competition adsorption of H_2O and CO on Cu(100), Cu(711) and Cu(1021) surfaces as the function of electrode potential. The dotted lines represent the crossover of competitive adsorption.

Supplementary Note 9: Atomistic Thermodynamics Approach

1. Idea Gas

The enthalpy of gas phase molecules is expressed as:

$$H(T) = E_{elec} + E_{ZPE} + \int_0^T C_p dT$$

Where E_{elec} is the electronic energy, E_{ZPE} presents the zero point energy and the last term is the integral over the constant pressure heat capacity (C_{p}).

Ideal gas entropy as a function of T and P can be expressed as a sum of translational (S_{trans}), rotational (S_{rot}), electronic (S_{elec}) and vibrational (S_{vib}) contributions with a pressure correction as:

$$S(T,P) = S(T,P^0) - k_B ln\frac{P}{P^0} = S_{trans} + S_{rot} + S_{elec} + S_{vib} - k_B ln\frac{P}{P^0}$$

Finally using the enthalpy and entropy, we find the chemical potential of the gas phase molecule (μ_{gas}) as:

$$\mu_{gas} = G(T,P) = H(T) - TS(T,P)$$

2. Harmonic Oscillator (HO) approximation

Assuming that the adsorbate has no real translational and rotational degree of freedom, we can treat all the 3N DOF of the adsorbates harmonically. Using this approximation, we can calculate the internal energy (U) and entropy of the adsorbate as follows:

$$U(T) = E_{elec} + E_{ZPE} + \sum_{i}^{harm\ DOF} \frac{\epsilon_i}{e^{\epsilon i/k_B T} - 1}$$

$$S = k_B \sum_{i}^{harm\ DOF} [\frac{\epsilon_i}{k_B T(e^{\epsilon i/k_B T} - 1)} - ln(1 - e^{-\epsilon i/k_B T})]$$

Where, ϵ_i are the harmonic energies for the adsorbate atoms.

The Helmholtz free energy (F) can hence be calculated as:

$$F(T) = U(T) - TS(T)$$

Assuming that the pV term in $H = U + pV$ is negligible, the Helmholtz Free energy can be used as an approximate for the Gibbs Free energy since $G \approx F$.

Supplementary Note 10: The choice of analytical approximation.

The flatness of the potential energy surface (PES) poses a significant challenge in microkinetic modeling. First, the reaction rates are highly sensitive to the reaction energy and barriers of the elementary steps. When the PES is flat, the differences in energy between various intermediates become minimal. This increases the difficulty in accurately determining the energy barriers for elementary steps (due to the possibly premature convergence of the electronic structure solver to the threshold of near-zero gradients), leading to significant uncertainty in the modeling results. Second, a flat PES implies that small perturbations can have disproportionately large effects on the reaction pathways and rates. The intrinsic error of DFT (~0.15 eV) makes the microkinetic models highly sensitive and unpredictable. In addition, in a flat PES, it is challenging to identify distinct reaction pathways. This lack of clarity can hinder the ability to map out the reaction mechanism accurately, which is crucial for microkinetic modeling. Thus, in this work, we considered the analytical approximation where the rate-determining step determines the overall activity to compare the reaction rate.

Reference

1. Gauthier, J.A. et al. Challenges in Modeling Electrochemical Reaction Energetics with Polarizable Continuum Models. *ACS Catal.* **9**, 920-931 (2018).
2. Peng, H. et al. The role of atomic carbon in directing electrochemical $CO_{(2)}$ reduction to multicarbon products. *Energy Environ. Sci.* **14**, 473-482 (2021).
3. Xiao, H., Goddard, W.A., 3rd, Cheng, T. & Liu, Y. Cu metal embedded in oxidized matrix catalyst to promote CO_2 activation and CO dimerization for electrochemical reduction of CO_2. *Proc. Natl. Acad. Sci. USA* **114**, 6685-6688 (2017).
4. Lian, Z., Dattila, F. & López, N. Stability and lifetime of diffusion-trapped oxygen in oxide-derived copper CO_2 reduction electrocatalysts. *Nat. Catal.* **7**, 401-411 (2024).
5. Garza, A.J., Bell, A.T. & Head-Gordon, M. Is Subsurface Oxygen Necessary for the Electrochemical Reduction of CO_2 on Copper? *J. Phys. Chem. Lett.* **9**, 601-606 (2018).
6. Fields, M., Hong, X., Nørskov, J.K. & Chan, K. Role of Subsurface Oxygen on Cu Surfaces for CO_2 Electrochemical Reduction. *J. Phys. Chem. C* **122**, 16209-16215 (2018).
7. Herzog, A. et al. Operando Raman spectroscopy uncovers hydroxide and CO species enhance ethanol selectivity during pulsed $CO_{(2)}$ electroreduction. *Nat. Commun.* **15**, 3986 (2024).
8. Timoshenko, J. et al. Steering the structure and selectivity of CO_2 electroreduction catalysts by potential pulses. *Nat. Catal.* **5**, 259-267 (2022).
9. Arán-Ais, R.M., Scholten, F., Kunze, S., Rizo, R. & Roldan Cuenya, B. The role of in situ generated morphological motifs and Cu(i) species in C_{2+} product selectivity during CO_2 pulsed electroreduction. *Nat. Energy* **5**, 317-325 (2020).
10. Mistry, H. et al. Highly selective plasma-activated copper catalysts for carbon dioxide reduction to ethylene. *Nat. Commun.* **7**, 12123 (2016).
11. Favaro, M. et al. Subsurface oxide plays a critical role in CO_2 activation by Cu(111) surfaces to form chemisorbed CO2, the first step in reduction of CO2. *Proc. Natl. Acad. Sci. USA* **114**, 6706-6711 (2017).
12. Scott, S.B. et al. Absence of Oxidized Phases in Cu under CO Reduction Conditions. *ACS Energy Lett.***4**, 803-804 (2019).
13. Lum, Y. & Ager, J.W. Stability of Residual Oxides in Oxide-Derived Copper Catalysts for Electrochemical CO2 Reduction Investigated with (18) O Labeling. *Angew. Chem. Int. Ed.* **57**, 551-554 (2018).
14. Zhao, Y. et al. Speciation of Cu Surfaces During the Electrochemical CO Reduction Reaction. *J. Am. Chem. Soc.* **142**, 9735-9743 (2020).
15. Vijay, S. et al. Unified mechanistic understanding of CO reduction to CO on transition metal and single atom catalysts. *Nat. Catal.* **4**, 1024-1031 (2021).
16. Chan, K. A few basic concepts in electrochemical carbon dioxide reduction. *Nat. Commun.* **11**, 5954 (2020).
17. Xu, A., Govindarajan, N., Kastlunger, G., Vijay, S. & Chan, K. Theories for Electrolyte Effects in CO_2 Electroreduction. *Acc. Chem. Res.* **55**, 495-503 (2022).
18. Resasco, J. et al. Promoter Effects of Alkali Metal Cations on the Electrochemical Reduction of Carbon Dioxide. *J. Am. Chem. Soc.* **139**, 11277-11287 (2017).
19. Ringe, S. et al. Understanding cation effects in electrochemical CO_2 reduction. *Energy Environ. Sci.* (2019).
20. Ringe, S. et al. Double layer charging driven carbon dioxide adsorption limits the rate of electrochemical carbon dioxide reduction on Gold. *Nat. Commun.* **11**, 33 (2020).
21. Wei, Z. & Sautet, P. Improving the Accuracy of Modelling $CO_{(2)}$ Electroreduction on Copper Using Many-Body Perturbation Theory. *Angew. Chem. Int. Ed.* **61**, e202210060 (2022).

22. Malkani, A.S. et al. Understanding the electric and nonelectric field components of the cation effect on the electrochemical CO reduction reaction. *Sci. Adv.* **6** (2020).
23. Monteiro, M.C.O. et al. Absence of CO_2 electroreduction on copper, gold and silver electrodes without metal cations in solution. *Nat. Catal.* **4**, 654-662 (2021).
24. Gao, D. et al. Activity and Selectivity Control in CO_2 Electroreduction to Multicarbon Products over CuO_x Catalysts via Electrolyte Design. *ACS Catal.* **8**, 10012-10020 (2018).
25. Li, C.Y. et al. In situ probing electrified interfacial water structures at atomically flat surfaces. *Nat. Mater.* **18**, 697-701 (2019).
