# Supplementary material for: Structure Sensitivity and Catalyst Restructuring for CO2 Electro-reduction on Copper
Source: Nat Commun. 2025 Apr 30;16:4064. doi: 10.1038/s41467-025-59267-3 (PMC12043938; doi:10.1038/s41467-025-59267-3)
Supplement: Supplementary file 2 — Description of Additional Supplementary Files [file 41467_2025_59267_MOESM2_ESM.docx]

**Description of Additional Supplementary Files**

Supplementary Data 1

Description: coordinate files for CO covered Cu (410), (430), (533), (711), (843) and (1021) surfaces

Supplementary Code 1

Description:

Supplementary Movie 1

Description:
